# Higher-Magnesium-Doping Effects on the Singlet Ground State of the Shastry–Sutherland SrCu_2_(BO_3_)_2_

**DOI:** 10.1021/acs.inorgchem.4c02398

**Published:** 2024-10-16

**Authors:** Lia Šibav, Žiga Gosar, Tilen Knaflič, Zvonko Jagličić, Graham King, Hiroyuki Nojiri, Denis Arčon, Mirela Dragomir

**Affiliations:** † Jožef Stefan Institute, Jamova cesta 39, Ljubljana 1000, Slovenia; ‡ Jožef Stefan International Postgraduate School, Jamova cesta 39, Ljubljana 1000, Slovenia; § Faculty of Mathematics and Physics, University of Ljubljana, Jadranska ulica 19, Ljubljana 1000, Slovenia; ∥ Research Institute, Institute for the Protection of Cultural Heritage of Slovenia, Poljanska cesta 40, Ljubljana 1000, Slovenia; ⊥ Physics and Mechanics, Institute of Mathematics, Jadranska ulica 19, Ljubljana 1000, Slovenia; # Faculty of Civil and Geodetic Engineering, University of Ljubljana, Jamova cesta 2, Ljubljana 1000, Slovenia; ∇ Canadian Light Source, 44 Innovation Blvd, Saskatoon, SK S7N 2V3, Canada; ○ Institute for Materials Research, Tohoku University, Katahira 2-1-1, Sendai 980-8577, Japan

## Abstract

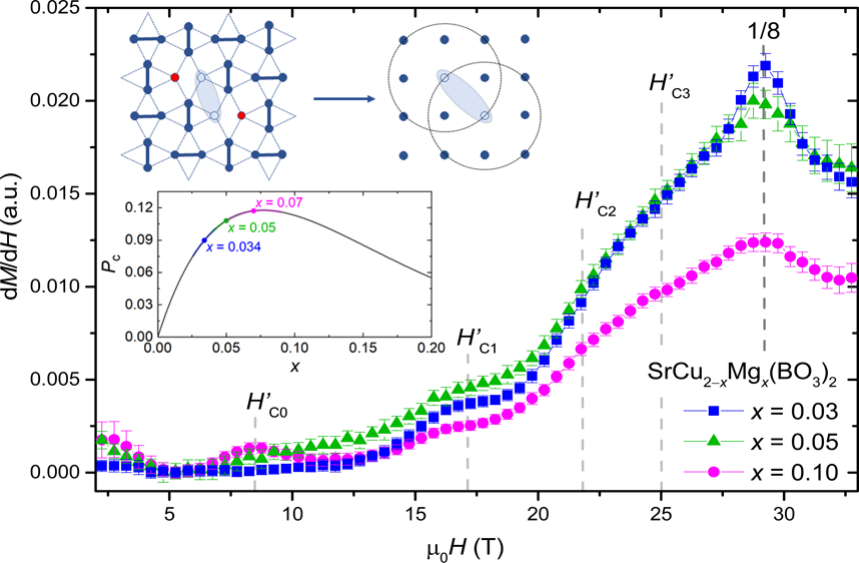

Doping of quantum antiferromagnets is an established approach to investigate the robustness of their ground state against the competing phases. Predictions of doping effects on the ground state of the Shastry–Sutherland dimer model are here verified experimentally on Mg-doped SrCu_2_(BO_3_)_2_. A partial incorporation of Mg^2+^ on the Cu^2+^ site in the SrCu_2_(BO_3_)_2_ structure leads to a subtle but systematic lattice expansion with the increasing Mg-doping concentration, which is accompanied by a slight decrease in the spin gap, the Curie–Weiss temperature, and the peak temperature of the susceptibility. These findings indicate a doping-induced breaking of Cu^2+^ spin-1/2 dimers that is also corroborated by X-band EPR spectroscopy that points to a systematic increase in the intensity of free Cu^2+^ sites with increasing Mg-doping concentration. Extending the Mg-doping up to nominal *x* = 0.10 yielding SrCu_1.9_Mg_0.1_(BO_3_)_2_, in the magnetization measurements taken up to 35 T, a suppression of the pseudo-1/8 plateau is found along with a clear presence of an anomaly at an onset critical field μ_0_*H’*_C0_ ≈ 9 T. The latter, absent in pure SrCu_2_(BO_3_)_2_, emerges due to the pairwise coupling of liberated Cu^2+^ spin-1/2 entities in the vicinity of Mg-doping induced impurities.

## Abstract 2

High-field magnetic susceptibility measurements of SrCu_2−̵*x*_Mg*x*(BO_3_)_2_ with nominal *x* = 0.10 up 35 T revealed a maximum in d*M*/d*H* at μ_0_*H* ≈ 9 T, indicating an impurity-driven spin configuration attributed to the stabilization of coupled Cu^2+^ moments liberated by Mg-doping. With increasing the nominal doping values, the probability for the occurrence of the next-nearest liberated Cu^2+^ moments reached a maximum at *x* ≈ 0.07.

## 1 Introduction

Quantum fluctuations, low dimensionality and geometric frustration are considered important ingredients for exotic states of matter such as quantum spin liquids^1,2^ or high-*T*_C_ superconductivity.^3^ In particular, low-dimensional materials with antiferromagnetic (AFM) interactions and coupled *S* = 1/2 moments are the most promising to experimentally explore the realization of such exotic states.^4−7^ In the last decades, transition metal oxides exhibiting such physics have been actively studied. A particular interest is devoted to the family of high-*T*_C_ cuprate superconductors where the observation of two-dimensional (2D) antiferromagnetism in Mott-insulating parent compounds led to the suggestion of an intimate correlation of the gapped spin excitations with unconventional superconductivity in charge-doped compounds.^8^ Following this discovery, numerous studies focused on other 2D spin systems with spin-gapped ground states.^9,10^ While the spin-singlet ground state is commonly found in one-dimensional (1D) or quasi-1D materials,^11−15^ there are only few examples of 2D spin-gapped systems^16−18^ due to their tendency of long-range ordering guided by the interlayer exchange interactions.

One rare example of a spin-gapped 2D material is strontium copper orthoborate, SrCu_2_(BO_3_)_2_.^18^ As the first experimental realization of the theoretical Shastry-Sutherland model,^19^ this frustrated 2D spin system contains spin dimers which are orthogonally coupled on a square lattice. In this model, the in-plane coupling of Cu^2+^ (*S* = 1/2) spins is described by the antiferromagnetic nearest neighbor (*NN*) coupling *J* and the next-nearest neighbor (*NNN*) coupling *J*’:
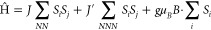
1

The last term in eq 1 is the Zeeman interaction with the external magnetic field, *B*. The model Hamiltonian has been successfully applied to SrCu_2_(BO_3_)_2_ to interpret, e.g., the results of electron paramagnetic resonance (EPR)^20−25^ and nuclear magnetic resonance (NMR) studies.^26−29^ Importantly, the *J’*/*J* ratio in eq 1 dictates the magnetic ground state.^19,30^ For *J’*/*J* < 0.69, the ground state is the exact dimer phase that is a product of singlets on Cu-dimers,^31^ while a gapped plaquette singlet state or a plaquette valence bond solid phase is found for 0.69 < *J’*/*J* < 0.86.^30,31^ For *J’*/*J* > 0.86, the Néel phase or the dimer valence bond solid phase is stabilized. Recently, a novel quantum spin liquid phase has been predicted to exist between the gapped plaquette-singlet phase and the antiferromagnetic phase.^32^ Experimentally, the *J′*/*J* ratio in SrCu_2_(BO_3_)_2_ was determined to be 0.68 (*J* = 100 K, *J’* = 68 K),^33^ with more recent reports claiming a slightly smaller value of 0.63(1).^34,35^ The value of *J*’/*J* positions SrCu_2_(BO_3_)_2_ to the exact singlet-dimer state of the Shastry-Sutherland phase diagram. However, it is also very close to the quantum critical point that separates the singlet-dimer state to other competing phases.^28^ This specific property of SrCu_2_(BO_3_)_2_ opens up an intriguing possibility to investigate the stability and behavior of a frustrated quantum antiferromagnet close to a quantum critical point.

The exact dimer phase of SrCu_2_(BO_3_)_2_ was first challenged in high-magnetic field experiments aiming to close the spin gap, Δ. The first estimate of a spin gap Δ of 19(1) K was from low-temperature magnetic susceptibility,^36^ but more precise values of 34(1) K were obtained from other experiments such as high-field EPR.^37^ Upon closing Δ with magnetic field, the presence of strong frustration is responsible for the Wigner-like crystallization of triplets at critical fields, giving rise to characteristic magnetization plateaus.^38^ Namely, the magnetization plateaus at fractional values of the saturated magnetization of 1/8 and 1/4 were initially observed.^18,36^ Later, other intermediate fractional plateaus were also reported such as 1/3^39^ and 1/9, 1/7, 1/6, 1/5, 2/9,^40^ adding to the extreme richness of the SrCu_2_(BO_3_)_2_ phase diagram. A very recent study showed evidence of a spin nematic phase or bound-state condensate in SrCu_2_(BO_3_)_2_ before the 1/8 plateau and beyond 21 T, which is understood as a condensate of bosonic Cooper pairs.^41^ Alternatively, the ground state of SrCu_2_(BO_3_)_2_ may be altered by means of physical pressure or chemical pressure through substitutions. Applying hydrostatic pressure on SrCu_2_(BO_3_)_2_^27,42,43^ was shown to induce two quantum phase transitions: (i) around 1.7 GPa, where the dimer singlet phase transitions to a plaquette singlet phase below 2 K, (ii) at 3–4 GPa, where another antiferromagnetic phase is realized below 4 K.^44^

On the other hand, the effects of chemical pressure or chemical-doping-induced impurities on the ground state of SrCu_2_(BO_3_)_2_ are less understood due to difficulties in chemical modifications of this compound. However, theoretical studies have predicted that altering the ground state of SrCu_2_(BO_3_)_2_ by chemical doping could result in exotic magnetic states such as quantum spin liquids or superconductors.^45,46^ In spite of these promising theoretical predictions, the literature on the experimental doping of SrCu_2_(BO_3_)_2_ is scarce^47−52^ as introducing dopants into the crystal structure of SrCu_2_(BO_3_)_2_ has been proven to be a very challenging process even for low doping concentrations.

The chemical substitutions on the Sr site, located between the Cu–O–B layers in the SrCu_2_(BO_3_)_2_ structure, could exert both chemical pressure on the Cu–Cu exchange pathways and charge imbalance through a different valence state of the dopant, affecting the Cu magnetic moment. Doping on the Sr site with Y, Na, La, Zn or Al led to a suppression of the spin gap in polycrystalline samples,^49^ but the gap was not fully closed and no superconductivity was observed. Single crystals of Na- and La-doped SrCu_2_(BO_3_)_2_ have also been grown by the optical floating zone method.^50^ Although the X-ray diffraction (XRD) measurements showed that these compounds are single-phase and their in-plane lattice parameter depends systematically on the dopant content *x*, a detailed structural and local-probe investigation such as EPR or NMR of these dopants is still missing. Moreover, a quantum spin liquid is anticipated to be realized by magnetic dilution–partially substituting the magnetic Cu^2+^ ions with nonmagnetic isovalent cations, which could break the spin dimers and establish a long-range quantum entanglement at the lowest temperatures.^47^ Magnetic dilution, i.e., Mg^2+^ substitutions on the Cu^2+^ site, has been proven to be chemically even more challenging and only a few reports exist on Mg-doped SrCu_2_(BO_3_)_2_. Previous studies focused on SrCu_2–*x*_Mg_*x*_(BO_3_)_2_ with nominal *x* ≤ 0.05 including measurements such as muon spin relaxation studies (μSR)^47^ magnetometry at high magnetic fields,^49^ and neutron scattering.^51^ Subtle changes in magnetism with Mg doping have been suggested. The μSR studies on single crystals of nominal *x* = 0.05^47^ revealed that Mg doping disrupts the formation of local singlets, as evidenced by the emergence of a third frequency shift branch attributable to the dopant effect. This third branch represents the case when the muon resides at a site in the superexchange path where a local singlet cannot form due to doping, leading to the conclusion that Mg-doping prevents the formation of some singlets both in and out of the Cu–B–O planes.

High-field magnetometry studies showed that impurities disrupt the formation of superstructures of bound triplet states, breaking them into smaller patches. This significant softening of the superstructures causes the smearing of the pseudo-1/*n* plateaus in the doped sample, with the expectation that these plateaus would be totally suppressed with increased doping. Further studies are desired to better understand the impurity-induced emergent states, to search for other possible novel phases at high fields, in particular on samples with higher Mg concentrations.

Neutron-scattering experiments have shown the appearance of new magnetic excitations into the singlet–triplet gap.^51^ High resolution time-of-flight neutron scattering measurements on single crystals of SrCu_2–*x*_Mg_*x*_(BO_3_)_2_ with nominal *x* = 0.05 Mg have shown that doping of the magnetic Cu^2+^ site with nonmagnetic, isoelectronic Mg^2+^ introduces new magnetic excitations into the singlet energy gap, and gives a finite lifetime to all three single-triplet excitations, while also substantially broadening the two-triplet bound state. However, there are no studies on samples with Mg concentrations *x* > 0.05. Moreover, there are no studies on the local structure around the dopants using local-probe magnetic resonance techniques such as EPR or NMR.

As the ionic radii of Cu^2+^ and Mg^2+^ are almost identical (∼0.57 Å) for a square planar coordination,^53^ the observed subtle and nonsystematic structural changes are not surprising. This renders also the determination of the true range of doping difficult. However, while Cu^2+^ can adopt both an octahedral and a square planar coordination, as in SrCu_2_(BO_3_)_2_, Mg^2+^ chemically prefers an octahedral coordination. Therefore, achieving a substantial concentration of Mg incorporated into the SrCu_2_(BO_3_)_2_ structure still remains a challenge. With the aim of pushing the doping level to higher values and addressing the robustness of the exact dimer phase, this paper reports a comprehensive study of Mg-doping of polycrystalline SrCu_2_(BO_3_)_2_, targeting the 10 mol % nominal doping concentration or *x* = 0.10 in SrCu_2–*x*_Mg_*x*_(BO_3_)_2_. To achieve such high doping levels, a different source of magnesium was used, namely Mg_2_CO_3_(OH)_2_ · 3.75 H_2_O, which is expected to increase the reactivity and help with Mg-incorporation. A successful substitution of Cu with Mg in the parent SrCu_2_(BO_3_)_2_ structure is proven by powder XRD and energy-dispersive X-ray spectroscopy. It is then shown that Mg-doping leads to a softening of the magnetism in SrCu_2_(BO_3_)_2_ as suggested by a systematic decrease in the spin gap, Δ, the Curie–Weiss temperature, θ, and the maximum of the magnetic susceptibility, *T*_max_. The effects of Mg-doping on the Cu^2+^ dimers are further evaluated with EPR spectroscopy. These measurements show that the EPR spectra broaden with Mg-doping due to the increase in the concentration of intrinsic paramagnetic dimer-free Cu^2+^ sites that are coupled to the spin dimers. Finally, the weak anomaly at an onset critical field of *H’*_C0_ ≈ 9 T, previously first observed by Shi et al.^48^ for their *x* = 0.05 Mg-doped SrCu_2–x_Mg_*x*_(BO_3_)_2_, is here investigated using high-field magnetization measurements up to 35 T. We find that this anomaly is more pronounced for *x* = 0.10 and its intensity systematically increases with Mg concentration. This work thus constitutes a systematic study of the effect of magnetic dilution on the magnetic properties of SrCu_2_(BO_3_)_2_ and opens alternative pathways toward higher substitutional doping of SrCu_2_(BO_3_)_2_.

## 2 Experimental Section

### 2.1 Materials and Methods

Pristine and Mg-doped SrCu_2_(BO_3_)_2_ polycrystalline samples SrCu_2–x_Mg_*x*_(BO_3_)_2_ with *x* being 0.03, 0.05, and 0.1, respectively, were synthesized by the traditional solid-state method and using boric acid as the boron source. The overall reactions are summarized in eqs 2 and 3:
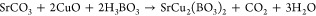
2
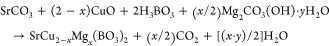
3

The starting materials were high-purity SrCO_3_ (Alfa Aesar, 99.994%), CuO (Aldrich, 99.99%) and H_3_BO_3_ (Alfa Aesar, 99.9995%). The starting material used as Mg-source was Mg_2_CO_3_(OH)_2_ · *y*H_2_O (*y* ≈ 3) (Alfa Aesar, 99.996%). The coefficient *y* in Mg_2_CO_3_(OH)_2_ · *y*H_2_O (*y* ≈ 3) was determined to be 3.75 by thermal gravimetric analysis. This precursor was stored and weighed in a MBraun glovebox under an argon atmosphere of <0.1 ppm of O_2_ and <0.1 ppm of H_2_O.

The starting materials were weighed in stoichiometric ratios and hand-homogenized in an agate mortar for an hour. The homogenized gray powder was then pressed into pellets with an 8 or 10 mm diameter with a force of 5 tonnes. The pellets were fired for multiple cycles with several intermediate grindings, following the thermal profile: 780 °C for 24 h and 810 °C for 24 h in air plus multiple 3- or 6-day cycles at 900 °C in O_2_ atmosphere, until no changes were noticed in the PXRD pattern. More details about this synthesis procedure can be found in reference.^54^

### 2.2 Characterization

#### 2.2.1 X-ray Powder Diffraction (PXRD)

The phase composition of the obtained samples was investigated by powder X-ray diffraction (PXRD) using a PANalytical X’Pert Pro powder diffractometer with CuKα_1_ radiation in 2θ range 10–120° with a step size of 0.016° and a counting time of 300 s per step.

Synchrotron powder X-ray diffraction data were also collected at the Brockhouse high energy wiggler beamline at the Canadian Light Source (CLS) using an area detector of a 200 × 200 μm pixel size, and a λ = 0.3502 Å radiation with Ni as calibrant. The powders were packed into Kapton capillaries and measured in 2θ range 1–26°. The structural refinements were performed with the Rietveld method using the program GSAS-II.^55^

#### 2.2.2 Scanning Electron Microscopy (SEM) and Energy-Dispersive X-ray Spectroscopy (EDS)

For the microstructural investigations, Mg-doped SrCu_2_(BO_3_)_2_ powders were embedded in an epoxy resin matrix polished and carbon coated using a sputter coater model Balzers SCD 050. The imaging and compositional analyses were performed using Thermo Fisher Quanta 650 ESEM equipped with an energy-dispersive X-ray spectrometer (Oxford Instruments, AZtec Live, Ultim Max SDD 65 mm^2^) and a field-emission-gun scanning electron microscope (FE-SEM; JEOL JSM-7600) further equipped with an energy-dispersive X-ray spectrometer (EDS; INCA Oxford 350 EDS SDD), electron backscatter diffraction (EBSD) and a wavelength-dispersive X-ray spectrometer (WDXS; INCAWave 500). The accelerating voltage was 20 kV.

#### 2.2.3 Magnetic Susceptibility

The magnetic susceptibility measurements were performed on an MPMS-XL-5 SQUID magnetometer from Quantum Design within the 1.9–300 K temperature range and an applied magnetic field of 1 kG. Powder samples were placed inside a plastic capsule, which was inserted into a standard straw sample-holder and closed with a plastic stopper at one end.

#### 2.2.4 High-Field Magnetic Susceptibility

High-field magnetization measurements were performed at 0.4 K in pulsed magnetic fields up to 35 T at Institute for Materials Research, Tohoku University. About 50 mg of polycrystalline Mg-doped SrCu_2_(BO_3_)_2_ was inserted into Kapton tubes, which were closed with stoppers from both sides. Measurements for each sample composition were performed multiple times. The reported results were binned in 0.5 T wide intervals and averaged between the data acquisition runs. The individual measurements are presented in the Supporting Information.

#### 2.2.5 Electron Paramagnetic Resonance Spectroscopy (EPR)

The continuous wave (CW) X-band electron paramagnetic resonance spectroscopy experiments were performed on a Bruker E500 spectrometer operating at 9.37 GHz, equipped with a Varian TEM104 dual cavity resonator, an Oxford Instruments ESR900 cryostat and an Oxford Instruments ITC503 temperature controller. Samples were measured at temperatures ranging from room temperature down to 4 K, typically applying 2 mW of microwave power and a 5 G modulation amplitude with the modulation frequency of 50 kHz. In a typical experiment, approximately 30 mg of powder sample, sealed in a standard 4 mm EPR quartz tubes under dynamic vacuum, were used for each measurement.

## 3 Results and Discussion

### 3.1 Powder X-ray Diffraction (PXRD)

**Figure 1:**
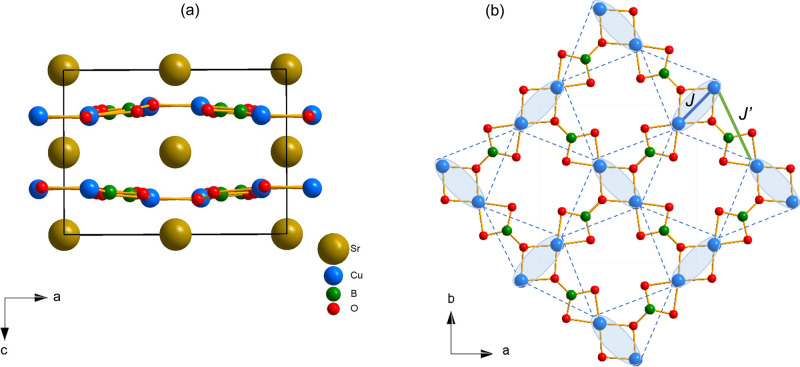
(a) The SrCu_2_(BO_3_)_2_ structure viewed along the *b*-axis. The Cu–B–O layers can be seen separated by Sr^2+^ ions. (b) View perpendicular to the Cu–B–O layer which shows the orthogonal Cu^2+^ spins coupled across the dimer bonds with the *NN* coupling *J*, represented by the blue solid line, and across the next-neighbor bonds with the *NNN* coupling *J’*, represented by the green solid line.

At room temperature, SrCu_2_(BO_3_)_2_ crystallizes in the *I*4̅2*m* space group–first reported in 1991 by Smith and Keszler,^56^ where distorted planar CuO_4_ units and rigid BO_3_ triangular groups form two-dimensional puckered layers of [Cu(BO_3_)_2_]^2–^ units at *z* ≈ 1/4 stacked along the [001] direction. These layers are separated by nonmagnetic Sr^2+^ ions located at *z* = 0, 1/2 – Figure 1a. The distortion from planar CuO_4_ rectangles is a consequence of sharing an O–O edge with a B atom while the other O–O edge is shared with a Cu atom. As a result of this distortion, the [Cu(BO_3_)_2_]^2–^ layers are corrugated. The Cu–O distances measured along the *c*-axis are larger than 3.25 Å. All the in-plane Cu^2+^ sites (*S* = 1/2) are crystallographically equivalent and a two-dimensional network of copper dimers is formed with the neighboring pairs oriented orthogonal to each other – Figure 1b. The first nearest neighbor (*NN*) Cu^2+^ pairs are distanced by 2.903 Å while the next-nearest neighbor (*NNN*) distance is at 5.133 Å.

**Figure 2:**
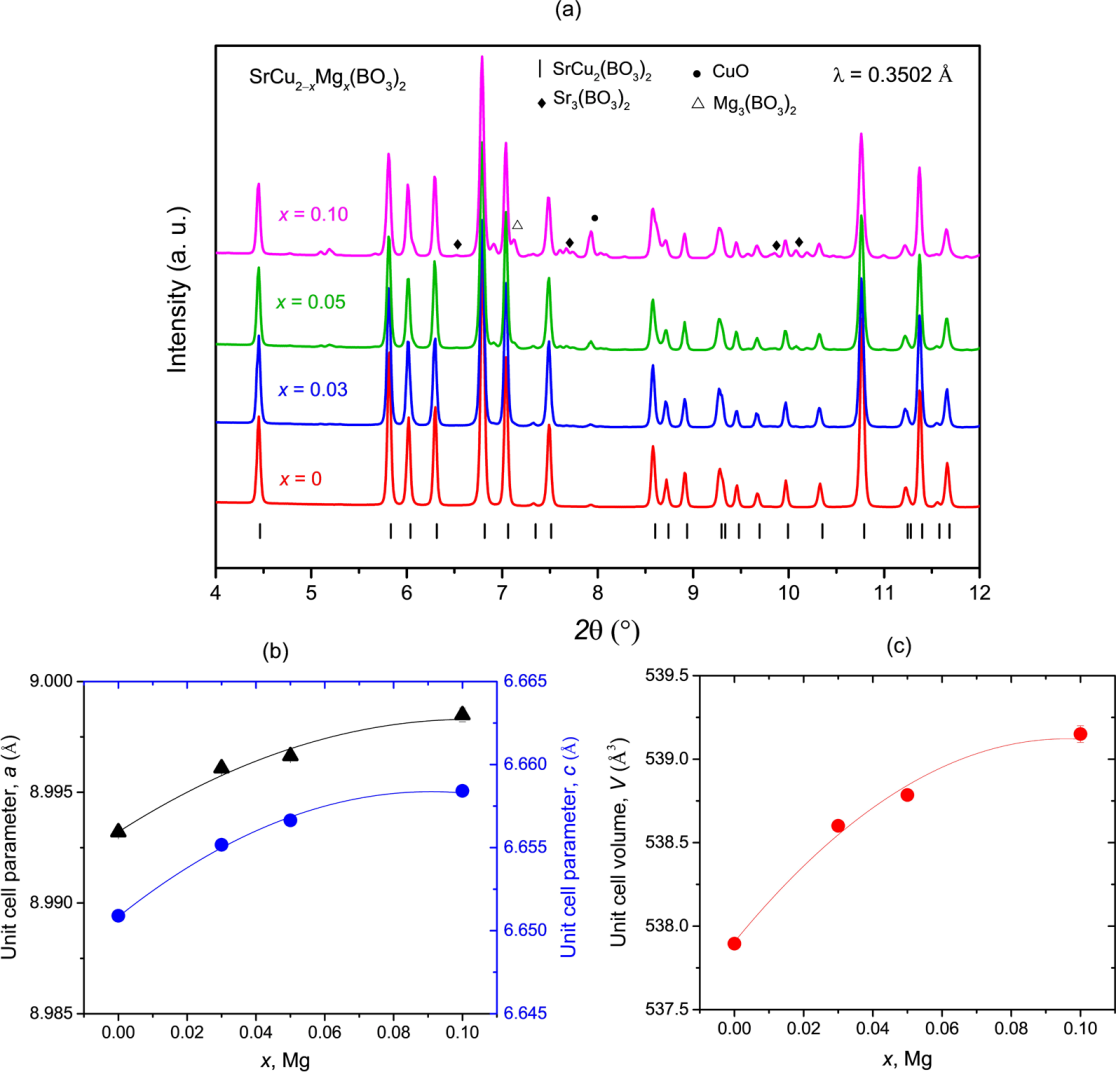
(a) A selected region of the room-temperature synchrotron PXRD patterns of SrCu_2–*x*_Mg_*x*_(BO_3_)_2_ with *x* = 0.03, 0.05 and 0.10, respectively. The formation of the dominant tetragonal SrCu_2_(BO_3_)_2_ phase can be seen in all patterns. Some CuO impurity (marked with solid circles), Mg-based secondary phases (open triangles) and Sr_3_(BO_3_)_2_ (solid diamonds) were also detected in the doped samples. A systematic increase in the unit cell parameters (b) and the unit cell volume (c) with Mg*-*doping is observed.

High-resolution X-ray powder diffractogram of the pristine SrCu_2_(BO_3_)_2_ shows the presence of the main SrCu_2_(BO_3_)_2_ phase, with traces of CuO – Figure 2a. We note that the minute presence of the persistent CuO impurity could not be avoided in powder samples, which is in agreement with previous reports.^50,56^ The Rietveld refinement analysis of the powder diffraction data yields *a* = 8.9932(1) Å and *c* = 6.6509(1) Å. These parameters are in very good agreement with the values reported in the literature, e.g., *a* = 8.998(1) Å and *c* = 6.654(1) Å from reference^50^ versus *a* = 8.991(1) Å and *c* = 6.660(3) Å in reference^56^ or *a* = 8.995(1) Å and *c* = 6.649(1) Å reported in references.^49,52^

Upon Mg-doping, the same two phases are consistently observed: the majority of the SrCu_2_(BO_3_)_2_ phase accompanied by the minor CuO impurity. However, the presence of CuO impurity grows with the nominal Mg concentration. Magnesium-based secondary phases are also present in the samples, as detected later with EDS analysis, with amounts increasing with the Mg concentration – Figure 2a.

The presence of these impurities underlines difficulties to incorporate Mg into the square planar coordination of Cu in the parent SrCu_2_(BO_3_)_2_ structure and suggests that the actual Mg-doping values are lower than the nominal values. Nevertheless, the current synthesis approach leads to Mg incorporation judging from a monotonic shift of the diffraction peaks toward lower 2θ values and a systematic increase of the unit cell parameters and volume with the increasing Mg-doping concentration – Figure 2b,**c**. The Rietveld refinement profiles are available in the Supporting Information, Figure S1. The unit cell parameters and unit cell volumes for SrCu_2–*x*_Mg_*x*_(BO_3_)_2_ with different *x* as obtained from Rietveld refinement analysis are directly compared in the Supporting Information, Table S1. Up to nominal *x* = 0.05, both *a* and *c* lattice parameters show a steady increase with Mg concentration, but then tend to saturate toward nominal *x* = 0.10. Specifically, the refined room temperature unit cell parameters increase to *a* = 8.9961(1) Å and *c* = 6.6552(1) Å for nominal *x* = 0.03, to *a* = 8.9966(2) Å and *c* = 6.6566(2) Å for nominal *x* = 0.05 and up to *a* = 8.9985(3) and *c* = 6.6584(3) Å for nominal *x* = 0.10, respectively **–**Figure 2b. Comparing these unit cell parameters with the available literature data for nominal *x* = 0.05, showing *a* = 8.994(1) Å, and *c* = 6.648(1) Å,^50^ this study shows a slightly larger Mg-doping induced lattice expansion for this doping concentration. However, the difference is still within the 3% tolerance of the unit cell volume regularly reported for undoped SrCu_2_(BO_3_)_2_.^49,52,56^ Compared to the parent pristine sample, the largest unit cell volume expansion of 0.23% is for the nominal *x* = 0.10. This increase is small and reflects the similarity in the ionic radius of Cu^2+^ and Mg^2+^ ions as well as the limited amount of the incorporated Mg^2+^. When comparing *x* = 0.05 and *x* = 0.10 samples, the relative change in both lattice parameters is extremely small and it amounts only about 0.002 Å. This suggests that the synthesis approach used in this study may have an upper limit for the effective Mg-doping into the SrCu_2_(BO_3_)_2_ structure up to these values.

A closer look at the structural details further reveals a systematic decrease in the Cu–Cu distance between Cu^2+^ pairs from 2.9205(9) Å for pristine SrCu_2_(BO_3_)_2_ (*x* = 0) to 2.9085(13) Å for SrCu_1.95_Mg_0.05_(BO_3_)_2_ (*x* = 0.05) and 2.9025(24) Å for SrCu_1.9_Mg_0.1_(BO_3_)_2_ (*x* = 0.10). A shorter Cu–Cu bond length (blue line labeled ***J*** in Figure 1b) should also reduce the Cu–O–Cu bond angle. The Cu–O–Cu bond angle indeed slightly decreases with Mg doping, from 98.9(1)° for the undoped sample (*x* = 0) to 98.5(2)° for *x* = 0.05, and 98.3(3)° for *x* = 0.10. As the Cu–O–Cu bond angle plays a substantial role in defining the effective superexchange interaction in strongly correlated transition metal oxides,^57^ this decrease in the bond angle tuned by the chemical pressure in the Mg-doped samples may lead to a small decrease in the strength of the intradimer antiferromagnetic exchange *J*. Meanwhile, a systematic increase in the interdimer distance Cu–Cu between the orthogonal dimers (green line labeled *J*’ in Figure 1b) from 5.1267(10) Å for SrCu_2_(BO_3_)_2_ (*x* = 0) to 5.1326(13) Å for SrCu_1.95_Mg_0.05_(BO_3_)_2_ (*x* = 0.05) and 5.1369(25) Å for SrCu_1.9_Mg_0.1_(BO_3_)_2_ (*x* = 0.10) is observed. This is consistent with the overall lattice expansion – Figure 2c.

### 3.2 Scanning Electron Microscopy (SEM) and Energy-Dispersive X-ray Spectroscopy (EDS)

The PXRD data clearly shows that the substitution of Cu^2+^ with Mg^2+^ in SrCu_2_(BO_3_)_2_ is possible, although not fully up to the nominal Mg concentrations. However, a detailed chemical analysis is mandatory to unambiguously confirm the incorporation of Mg and to obtain more quantitative information on the actual dopant concentration and distribution within the matrix as well as the morphology of the samples. For this reason, SEM and EDS analyses were carried out next. From the backscattered electron images, the lamellar SrCu_2_(BO_3_)_2_ matrix can be observed along with the presence of darker and lighter zones, which suggest the presence of impurities containing lighter and heavier elements, respectively. The lighter zone corresponds to the CuO impurity thus corroborating the PXRD results in Figure 2. The darker zones are attributed to the Mg-rich regions in the sample–more details on this can be found in the Supporting Information, Figure S2.

**Figure 3:**
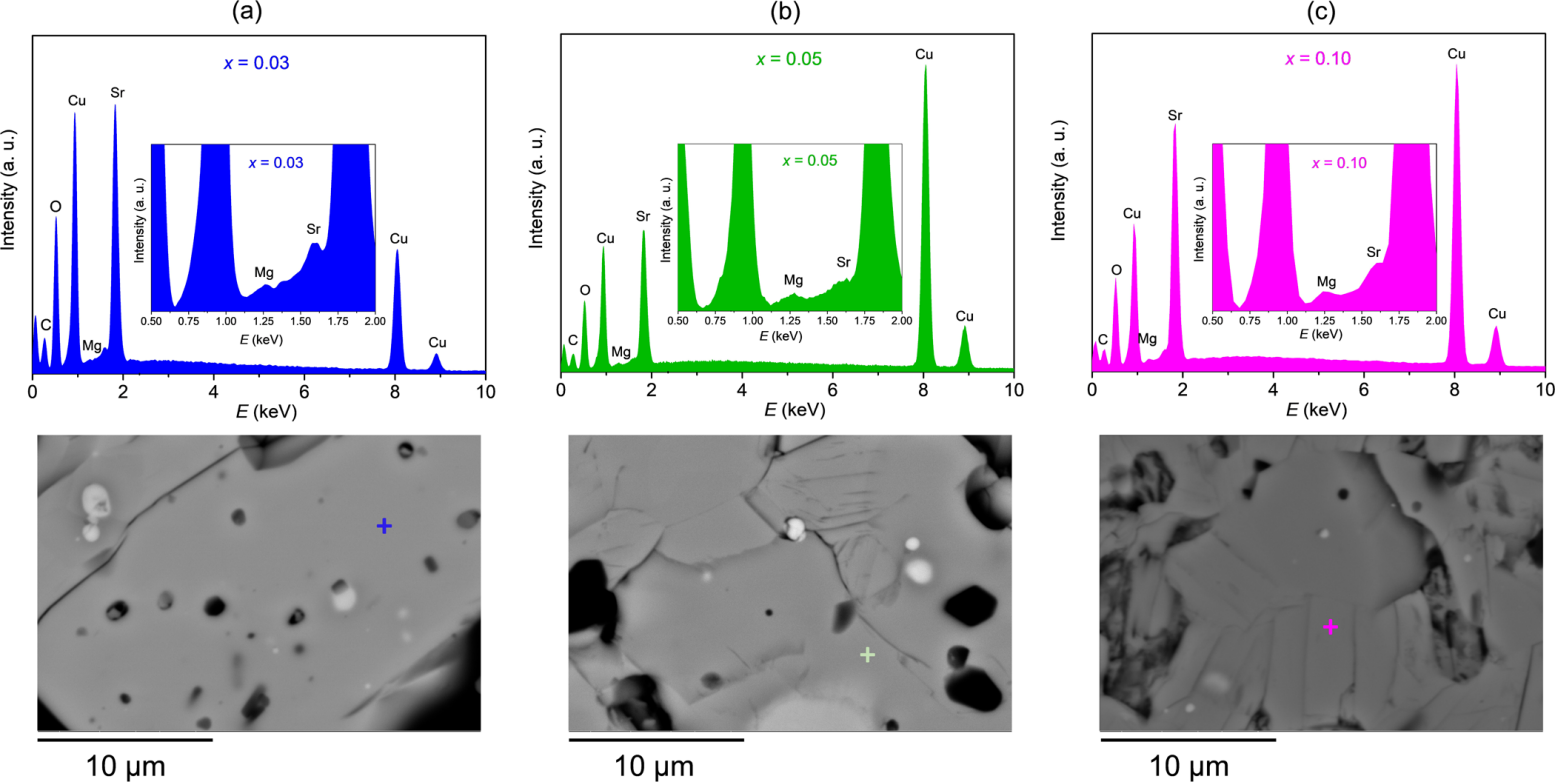
Representative EDS point analysis spectra collected on the matrix for SrCu_2–*x*_Mg_*x*_(BO_3_)_2_ with nominal *x* = 0.03 (a), *x* = 0.05 **(b),** and *x* = 0.1 (c). The corresponding SEM images can be seen below the EDS spectra. Colored squares mark the regions which EDS spectra were collected from.

Representative EDS point analysis spectra collected on ∼35 spots on SrCu_2*–x*_Mg_*x*_(BO_3_)_2_ samples with nominal *x* = 0.03, 0.05, and 0.10 and the corresponding SEM images are summarized in Figures 3a–c. The line at 1.25 eV in the EDS spectrum is the hallmark of the Mg emission and should thus be taken as a semiquantitative measure for the presence of Mg in the sample – Table 1. From the EDS point analysis, a clearly distinguished Mg emission peak is visible in all cases and thus unambiguously confirms the presence of Mg in the matrix for all three doping concentrations. Although the average semiquantitative values yield an approximate *x* = 0.07 for the nominal *x* = 0.10 sample, a clear trend showing an increase in *x* with the nominal doping concentrations is observed–see Table 1. The EDS point analysis also reveals a saturation of the Mg concentration that is successfully incorporated into the parent SrCu_2_(BO_3_)_2_ structure, which is in qualitative agreement with the conclusions drawn from PXRD in Figure 2b,c.

**Table 1:** Average Semiquantitative Atomic % Values of O, Mg, Cu and Sr together with the Mg/Cu, Mg/Sr and Cu/Sr Ratios^a^

**Element**	**Sample, SrCu**_**2–***x*_**Mg**_*x*_**(BO**_**3**_**)**_**2**_					
	*x* = 0.03		*x* = 0.05		*x* = 0.10	
	**Actual**	**Nominal**	**Actual**	**Nominal**	**Actual**	**Nominal**
	**Concentration, at %**					
**O**	63(2)	66.67	57(7)	66.67	57(7)	66.67
**Mg**	0.4(1)	0.33	0.6(1)	0.54	0.8(3)	1.11
**Cu**	24(2)	21.89	27(4)	21.67	28(4)	21.11
**Sr**	12.6(4)	11.11	15(3)	11.11	14(3)	11.11
**Sum, %**	100.0(2)	100.0	99.6(4)	100.0	99.8(3)	100.0
						
**Mg/Cu**	0.016	0.015	0.02	0.025	0.03	0.053
**Mg/Sr**	0.030	0.03	0.04	0.05	0.06	0.1
**Cu/Sr**	1.94	1.97	1.8	1.95	2.0	1.9
						
	**Concentration,** mol %					
**Mg**	3.4(8)	3.0	5(1)	5.0	7(2)	10.0

a These results were obtained from ∼35 different SEM-EDS point analyses on SrCu_2–*x*_Mg_*x*_(BO_3_)_2_ samples with *x* = 0.03, 0.05, and 0.10. The semi-quantifications were performed excluding boron as it was not possible to detect such a light element.

The presence of CuO and Mg-based secondary phase impurities observed in PXRD as well as in SEM stem from the partial incorporation of Mg into the parent SrCu_2_(BO_3_)_2_ structure.

The actual relative percentages of these impurities were difficult to calculate since a structural information file for the Mg_2_(BO_3_)_2_ phase is missing. However, we roughly estimated that the amount of impurities is below 1 wt % for Sr_3_(BO_3_)_2_ and below 2 wt % for CuO. The difficulty in reaching higher Mg concentrations in the SrCu_2_(BO_3_)_2_ lattice could be linked to the low mobility of the Mg^2+^ ions, which can be rationalized based on the fundamental diffusion mechanism and theory.^58^ The atomic diffusivity depends on the composition, temperature and pressure. In our case, the low SrCu_2_(BO_3_)_2_ decomposition temperature of ∼950 °C limits the maximum annealing temperatures to 900 °C. At this temperature, Mg^2+^ diffusion processes are slow, with diffusion constant values between 10^–14^–10^–11^ m^2^ s^–1^ which severely limits the possibility for successful Mg-doping.

### 3.3 Magnetic Susceptibility

**Figure 4:**
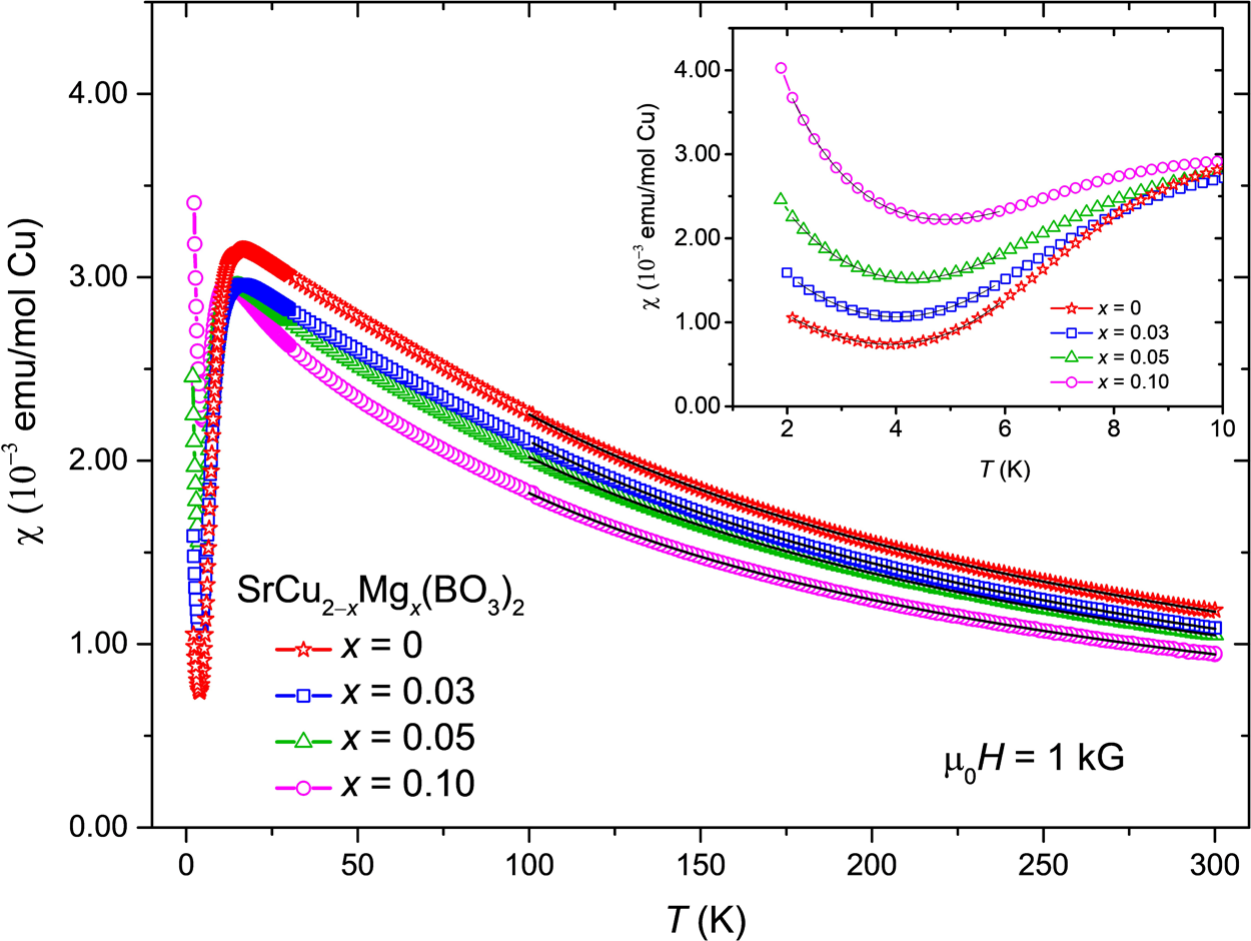
Magnetic susceptibility curves measured at μ_0_*H* = 1 kG for SrCu_2–*x*_Mg_*x*_(BO_3_)_2_ powders with *x* = 0, 0.03, 0.05, and 0.10 nominal doping concentration. The inset shows the expanded low-temperature 1.9–10 K region where the upturn in the magnetic susceptibility can be clearly seen to increase with Mg concentration. Black lines represent the fitted data with eq 4 for the high-*T* regime, 100–300 K, and using eq 5 for the low-*T* region, 2–6 K – here θ′ was fixed at −0.75 K.

Next, an investigation of the Mg-doping effects on the magnetic properties is presented. The magnetic susceptibilities, χ = *M*/*H*, measured for undoped and Mg-doped SrCu_2_(BO_3_)_2_ powders in the 1.9–300 K temperature range are compared in Figure 4. All samples generally show a qualitatively similar behavior: at high temperatures, χ follows the Curie–Weiss law, reaching a maximum at around ∼15 K, followed by a rapid suppression upon further cooling before it starts to increase again at the lowest temperatures. In order to quantitatively compare all samples, a fit of χ to a Curie–Weiss law in the high-temperature range of the data, between 100–300 K, is performed – eq 4:
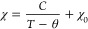
4Here *C* denotes the Curie constant, θ is the Curie–Weiss temperature, while χ_0_ represents a small temperature-independent diamagnetic contribution of the ion cores and/or the remnant signal of the sample holder. For the parent SrCu_2_(BO_3_)_2_ sample, the fit to eq 4 yields a θ = −135(1) K, which is in excellent agreement with the literature data and can be described with the Shastry-Sutherland model given in eq 1. For Mg-doped samples, a systematic and quite pronounced reduction in θ is observed — Table 2: θ decreases to −129(1) K and then to −110(1) K for nominal *x* = 0.05 and 0.10, respectively. Although one has to take these values with a grain of salt, the systematic reduction in θ with Mg-content implies a softening of the antiferromagnetic exchange interactions driven by the small structural changes observed in PXRD in Figure 2.

**Table 2:** Variation of Spin Gap, Δ, Extracted from Low-Temperature Susceptibility Fits in the Range 1.9–6 K, the *T*_max_, Which Is the χ Maximum Temperature, the Curie Constant C’ for the Low-Temperature Upturn in χ (for the Optimal Fit, a θ′ = −0.5 K Was Used) and the Estimated Concentrations of Unpaired Cu^2+^ Moments, and the Curie–Weiss Temperature, θ, Extracted from Curie–Weiss Fits of the Susceptibility Data in the Temperature Interval 100–300 K, for Different Mg-Doping Concentrations

Sample/Nominal *x*	Δ [K]	*T*_max_ [K]	C*’* (low-T, 1.9–3.5 K) in emu K/mol Cu	Estimated fraction of impurities / free Cu spins in %	Estimated Mg, in mol % / actual *x*	High-*T* fit, θ [K]
SrCu_2_(BO_3_)_2_	26.1(2)	16.6(2)	3.6(1) · 10^–3^	0.95(1)		–135(1)
*x = 0*						
SrCu_1.97_Mg_0.03_(BO_3_)_2_	22.0(2)	16.2(3)	5.2(2) · 10^–3^	1.37(4)	2.7(1)	–136(1)
*x* = 0.03						
SrCu_1.95_Mg_0.05_(BO_3_)_2_	19.6(2)	14.8(2)	7.2(2) · 10^–3^	1.9(1)	3.9(1)	–129(1)
*x* = 0.05						
SrCu_1.9_Mg_0.1_(BO_3_)_2_	15.9(4)	12.2(1)	10.7(2) · 10^–3^	2.85(5)	5.7(1)	–110(1)
*x = 0.10*						

It can be further noticed that the temperature at which χ(*T*) has a maximum, *T*_max_, also decreases from 16.6(2) K for the undoped sample to 12.2(1) K for the nominal *x* = 0.10 sample. This parallels the reduction in θ discussed above and is also consistent with the softening of antiferromagnetic exchange interactions.

Similarly, the introduction of nonmagnetic Mg^2+^ ions into the lattice affects also the low-temperature magnetic susceptibility.

Magnetic susceptibility data, as shown in the inset of Figure 4, show a characteristic Curie upturn at low temperatures, which is a hallmark of unpaired Cu^2+^ ions. In an attempt to quantitatively analyze χ(*T*) at low temperatures, we assume that it has three contributions^49^ – a dominant thermally activated contribution from the spin-dimer lattice characterized by the spin-gap Δ, a Curie–Weiss contribution from the unpaired Cu^2+^ moments in the lattice introduced by Mg-doping and quantified by their Curie constant C’, and the same temperature-independent contribution *χ*_*0*_ that was considered also in the high-temperature analysis:
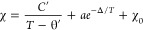
5

In these fits, a very small Curie–Weiss temperature θˈ = −0.5 K was fixed for optimal fitting. This minute θˈ probably accounts for weak residual interactions between orphan Cu^2+^ moments. Remarkably, just like θ and *T*_max_, the effective spin gap also decreases with Mg-content from 26.1(2) K for *x* = 0 to just 15.9(4) K for the nominal *x* = 0.10 sample, respectively. It should be stressed that the extracted Δ for the undoped sample matches the value from the literature where the same modeling was applied.^49^ Moreover, for nominal *x* = 0.05, a spin gap of 19.6(1) K is obtained which is in good agreement with the value reported in reference^47^ for the same nominal doping concentration.

Although the presented analysis of χ for the spin-dimer phase may not yield exact values for the different magnetic parameters, the systematic decrease in θ, *T*_max_ as well as in Δ – Table 2 – clearly points to a detrimental effect that Mg-doping has on the quantum magnetism of SrCu_2_(BO_3_)_2_. The minute structural changes of the Cu^2+^ dimer lattice in the Mg-doped samples – Figure 2 – are probably not sufficient to adequately change the *J’*/*J* ratio and trigger the transition to a plaquette-singlet, quantum spin liquid or Néel states as the spin gap remains open for all Mg concentrations. Yet, these structural changes are sufficient to fine-tune the intradimer and/or interdimer exchange interactions and thus to affect the extracted susceptibility parameters.

It is very intriguing that these changes occur concomitantly with the enhanced low-temperature Curie upturn in χ (for the full analysis see the Supporting Information, Tables S2 and S3). This upturn cannot be attributed to any of the CuO and Mg-based secondary impurity phases detected in PXRD, as none of them is expected to show a similar paramagnetic contribution. We therefore conclude that the low-temperature upturn in χ is in fact intrinsic to Mg-doped SrCu_2_(BO_3_)_2_ and is associated with the intrinsic lattice defects emerging when the nonmagnetic substitutional Mg^2+^ ions break the Cu-spin dimers. This conclusion is further supported by low temperature X-band EPR data (*vide infra*).

In order to quantify the paramagnetic defects in the lattice introduced by Mg-doping, the low-temperature Curie constant *C’* is calculated from Curie–Weiss fits in the temperature interval 1.9 K – 3.5 K and listed in Table 2. The individual fits are presented in the Supporting Information, Figure S3. A systematic increase in the *C*’ constant with Mg concentration is observed and thus the estimated fraction of impurities also increases with Mg concentration. Note that a similar Curie upturn is found already in the undoped powder sample for which the calculated Cu^2+^-impurity concentration corresponds to ∼0.95%. Such concentration is comparable to the literature data for powder samples.^36^ Moreover, for the nominal *x* = 0.03, the low-temperature upturn in doped samples is comparable with the literature.^48^ However, for the highest nominal *x* = 0.05 and 0.10, *C’* significantly exceeds literature values thus implying that our approach to Mg doping allows for higher Mg-concentrations in the SrCu_2_(BO_3_)_2_ lattice.

### 3.4 X-Band Electron Paramagnetic Resonance Measurements (EPR)

**Figure 5:**
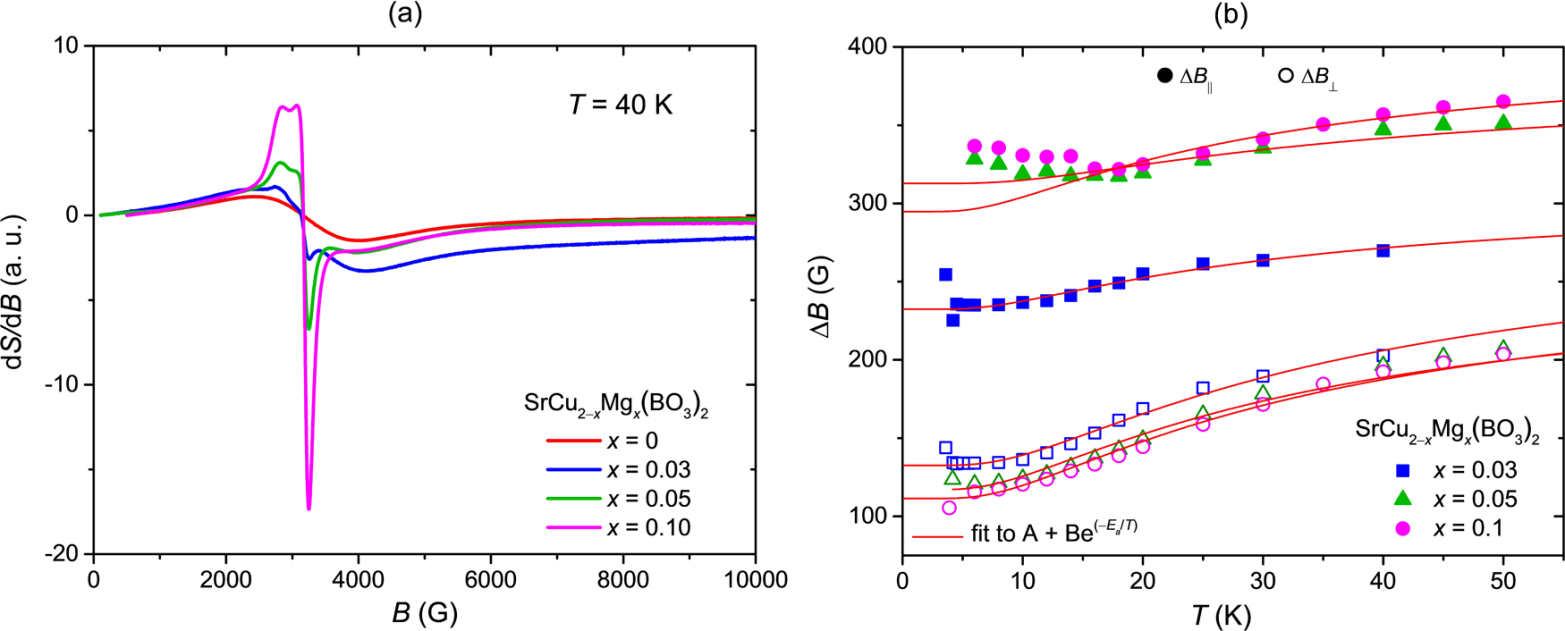
(a) The comparison of X-band EPR spectra of undoped SrCu_2_(BO_3_)_2_ and Mg-doped SrCu_2_(BO_3_)_2_ measured at 40 K. The EPR spectra of Mg-doped SrCu_2_(BO_3_)_2_ powders clearly show the presence of intrinsic Cu moments from broken Cu^2+^ dimers, represented by the sharp component with an axial g-factor anisotropy. (b) The temperature behavior of X-band EPR line width data for low-temperature impurity peaks in the *ab*-plane, (Δ*B*_⊥_) and along the *c*-axis-plane (Δ*B*_∥_) for SrCu_2–*x*_Mg_*x*_(BO_3_)_2_ with nominal*x* = 0, 0.03, 0.05, and 0.10. Solid lines are fits to the empirical A + B exp(−*E*_a_/*T*) expression yielding a thermally activated energy *E*_a_ ≈ 30 K for all sample compositions.

In the next step, a local probe technique, X-band EPR is employed to investigate the intrinsic Cu^2+^ defects that are created in the lattice upon Mg-doping. While EPR studies have been previously reported for undoped SrCu_2_(BO_3_)_2_ in the form of single crystals or powders,^20−25^ there are, however, no reports on EPR spectroscopy studies on doped SrCu_2_(BO_3_)_2_ yet. For Mg-doped samples, high temperature EPR spectra are still dominated by the well-known signal of the dimer lattice – Supporting Information, Figure S4. This signal broadens upon cooling due to the development of spin correlations and its intensity approximately mimics the temperature dependence of χ. The main broad signal almost completely disappears below 10 K, i.e., when *T* < Δ. At low temperatures, another signal with an axial *g*-factor anisotropy becomes visible – Supporting Information, Figure S4. While it is barely detectable for undoped SrCu_2_(BO_3_)_2_, it becomes significantly more pronounced in Mg-doped samples. Its presence is especially evident in the nominal *x* = 0.10 sample, where it dominates the spectrum already at 40 K as shown in Figure 5a, and can still be traced up to room temperature – Supporting Information, Figure S4(d). The intensity of this EPR component increases with the decrease in temperature and with increasing Mg-doping concentration, as it is shown in Supporting Information, Figure S5(d). This parallels the low-temperature dependence of the magnetic susceptibility and it is thus concluded that both contributions have the same origin–the intrinsic Cu moments from broken Cu^2+^ dimers.

The EPR spectra are thus simulated to a sum of two components: a broad Lorentzian line with a large line width Δ*B*_1/2_ that considers the main signal of the dimer lattice and the low-temperature component with an axial *g*-factor anisotropy characterized by *g*_∥_ and *g*_⊥_ as the two *g*-factor eigen-values along the *c-*axis and in the *ab*-plane crystallographic directions. In the latter case, a similar uniaxial anisotropy in the line width with two values for the line width Δ*B*_∥_ and Δ*B*_⊥_, respectively, is also assumed. For the main component at *g* = 2.095, Δ*B*_1/2_ ≈ 1400 G at room temperature is nearly identical for undoped as well as Mg-doped samples – Supporting Information, Figure S5a, and is consistent with the values determined for undoped SrCu_2_(BO_3_)_2_ powders from the literature.^20^

On cooling, Δ*B*_1/2_ initially monotonically increases in all samples, showing a similar temperature dependence that has also been previously reported for undoped SrCu_2_(BO_3_)_2_. However, below 40 K, we find a more significant increase in Δ*B*_1/2_ for nominal *x* = 0.05 and 0.10 samples when compared to *x* = 0 or undoped SrCu_2_(BO_3_)_2_ – Supporting Information, Figure S5a. This indicates the emergence of internal fields in doped samples that provide additional broadening mechanism associated with the presence of intrinsic impurities. The low-temperature “impurity” EPR component can be described in all samples with similar EPR parameters: *g*_∥_= 2.38, *g*_⊥_ = 2.008 – Supporting Information, Figure S5c, and a similarly pronounced line width anisotropy Δ*B*_∥_ ≈ 320 G and Δ*B*_⊥_ ≈ 120 G – Figure 5b.

Below 50 K, Δ*B*_∥_ and Δ*B*_⊥_ start to decrease with the decreasing temperature. This temperature dependence can be empirically described by a thermally activated mechanism, Δ*B* ∝ exp (−*E*_a_/*T*) – Figure 5b, with the activation energy *E*_a_ ≈ 30 K for all cases. The extracted value for *E*_a_ is very similar to the spin-gap values from spin susceptibility measurements, which suggests that these “impurity” spins are directly detecting the spin dynamics of the nearby unbroken Cu spin dimers.

The spins contributing to this low-temperature signal are thus indeed intrinsic Cu^2+^ defects from Mg-broken spin dimers sitting in the [Cu(BO_3_)]^−^ layers. Mg-doping is thus indeed successful and creates intrinsic Cu^2+^ defects that couple to the parent dimer lattice and effectively probe its spin dynamics.

### 3.5 High Magnetic Field Measurements

**Figure 6:**
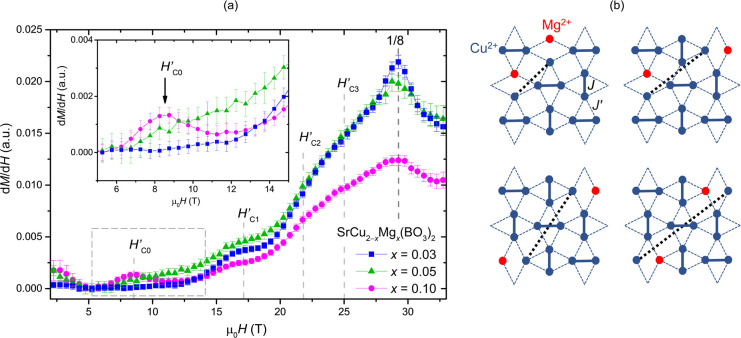
(a) High-field magnetic susceptibility measurements for SrCu_2–*x*_Mg_*x*_(BO_3_)_2_ with nominal *x* = 0.03, 0.05, and 0.10 powders in the region up to the pseudo-1/8 plateau. The critical field notations are termed after reference.^48^ The presented measurements are a result of an average of multiple pulsed experiments. The data shown here is the binned average of the individual measurements with a bin interval of 0.5 T - see Supporting Information, Figure S7 for individual results. (b) Special 2-impurity configurations of dimer-free Cu^2+^*S* = 1/2 spins remaining from Mg-magnetic dilution and coupled at distances beyond intradimer Cu^2+^ bonds. The anomaly at μ_*0*_*H*’_C0_*≈* 9 T highlighted in the inset of (a) is due to such next-nearest Cu^2+^ impurity pairs.

Variations in the parameters extracted from χ and X-band EPR with the increasing concentration of intrinsic defects may influence the magnetization plateaus and stabilize some novel states. Therefore, next, the magnetization curves, *M*(*H*), to fields up to 35 T for SrCu_2–*x*_Mg_*x*_(BO_3_)_2_ with nominal *x* = 0.03, 0.05, and 0.10 samples at *T* = 0.4 K are reported. The onset of different phases that are stabilized as a function of magnetic field are best observed in d*M*/d*H* plots – Figure 6a. In d*M*/d*H*, a plateau and a step appear as a dip and a peak, respectively. The dip can be seen as a kink when there is another increasing d*M*/d*H* component. In Figure 6a, it can be seen that d*M*/d*H* displays a peak at μ_0_*H*_C4_ = 29 T that corresponds to the onset of pseudo-1/8 plateau, analogous to the 1/8 plateau found in undoped SrCu_2_(BO_3_)_2_.^36^ We also found a reduction of d*M*/d*H* related to the 1/8 plateau phase in higher field site. With increasing Mg-doping, the peak slightly shifts toward lower fields and, most importantly, it is severely suppressed. This is consistent with expectations presented in reference (48) that the pseudo-1/*n* plateaus would be suppressed with increasing doping. The anomalies in d*M*/d*H* at μ_0_*H’*_C3_ ≈ 25.0 T and μ_0_*H’*_C2_ ≈ 21.7 T are less prominent in the present measurements, presumably because of the powder nature of the studied samples. According to reference,^48^*H’*_C2_ corresponds to localized bound states of triplets, whereas *H’*_C3_ is the anomaly of the localized bound states with extra localized triplets in the vicinity. The bump in d*M*/d*H* at μ_0_*H’*_C1_ ≈ 17.1 T is observed for all the Mg-doping concentrations. Following iPEPS calculations from reference (48), this anomaly appears upon doping when two magnesium impurities break nearest-neighboring dimers. The two liberated Cu^2+^*S* = 1/2 spins couple to a singlet that converts to a triplet at *H’*_C1_. Strong coupling of these pairs of Cu^2+^ liberated spins renders them unobservable in X-band EPR spectra. Interestingly, for nominal *x* = 0.10, this anomaly appears the least pronounced. The most important result of these measurements is the observance of a clear maximum in d*M*/d*H* at μ_0_*H* ≈ 9 T. The scaling of the d*M*/d*H* maximum at *H’*_C0_ with the intrinsic defect concentration determined from magnetic susceptibility and “impurity-peak” X-band EPR intensity suggests that liberated Cu^2+^ moments must contribute to the stabilization of novel impurity-driven spin configurations. Previously, hints of an extra magnetic structure stabilized at this field were deduced from tunnel diode oscillator magnetic susceptibility measurements and were attributed to the stabilization of the coupled Cu^2+^ moments liberated by Mg-doping on the next-nearest (or even more distant) dimers.^48^ Four examples of possible arrangements of pairs of liberated Cu^2+^*S* = 1/2 spins are represented in Figure 6b. Due to the longer exchange pathways, the coupling of liberated Cu^2+^ spins is weaker for these configurations and thus the gap to the excited triplet state closes at lower fields than in case of nearest-neighboring liberated spins.

We thus next calculate the probability for the occurrence of next-nearest liberated Cu^2+^ moments. For this purpose, we make an approximation where we substitute the spin dimer lattice with a square lattice, where each lattice site represents the center of a nonmagnetic spin dimer – Supporting Information, Figure S6(a) and (b). When two Mg impurities locate on two next-nearest dimers, this translates to two Cu^2+^ moments on next-nearest sites of the square lattice – Supporting Information, Figure S6(c) and (d). Following reference (59), we calculate the probability doping dependence *P*_c_(*x*) for such configurations, shown in Supporting Information, Figure S6(e). These results show, that with increasing doping values, the probability for the next-nearest configuration of liberated Cu^2+^ moments initially increases and reaches a maximum at nominal *x* ≈ 0.07. This analysis is thus in agreement with the observed increase of the d*M*/d*H* maximum at *H’*_C0_ and provides an additional support for the origin of *H’*_C0_.

The X-band EPR data discussed above may provide an extra insight into such next-nearest neighboring configurations of coupled Cu^2+^ spins. At elevated temperatures, liberated Cu^2+^ spins relax mainly via nearby triplet excitations thermally activated across the spin gap Δ = 30 K – as a result, Δ*B* follows the thermally activated temperature dependence as shown in Figure 5b. However, at temperatures well below Δ, this broadening mechanism becomes less efficient and thus spin correlations between Cu^2+^ moments on next-nearest broken dimers provide an additional broadening mechanism. This is especially pronounced in the samples with nominal *x* = 0.05 and 0.10, where Δ*B*_∥_ starts to increase again below ∼15 K, which roughly corresponds to a critical field *H’*_C0_. A combined high-field magnetization study together with X-band EPR data therefore supports the picture of coupled Cu^2+^ moments on nearest and next-nearest broken dimers as derived from iPEPS computations in reference (48).

## 4 Conclusions

This work shows that it is possible to push the Mg-doping boundary to higher values and thus tackle the phase diagram of SrCu_2_(BO_3_)_2_ over broader parameter space. It is demonstrated that by changing the source of Mg during the synthesis, the doping is pushed to an upper nominal limit of nominal *x* = 0.10 in SrCu_2–*x*_Mg_*x*_(BO_3_)_2_. This large concentration of Mg as dopant has not been reported before, as it is notoriously difficult to introduce Mg impurities into SrCu_2_(BO_3_)_2_ due to low Mg^2+^ diffusion constants at applied reaction temperatures. At these higher Mg-doping levels more pronounced structural changes in Mg-doped samples are observed: the unit cell parameters and volume show a systematic increase with the increasing doping concentration. However, the incorporation of Mg in the SrCu_2_(BO_3_)_2_ structure does not equal the nominal Mg concentration and even tends to saturate at lower values according to PXRD and SEM-EDS analyses. Nevertheless, the achieved Mg doping concentrations are already sufficient to affect the magnetic properties as expressed by a significant and systematic reduction of the spin gap, the Curie–Weiss temperature and the magnetic susceptibility maximum temperature with the increasing dopant concentration. These measurements are thus consistent with the breaking of Cu^2+^ dimers by magnetic dilution and are further corroborated by X-band EPR spectroscopy. Finally, reaching higher Mg^2+^ doping levels allows the detection of the μ_0_*H’*_C0_ ≈ 9 T anomaly more clearly. This anomaly is indicative of special configurations of dimer-free Cu^2+^*S* = 1/2 spins remaining from magnetic dilution and coupled at distances beyond the intradimer Cu^2+^ bonds.^48^ While the presented data on powder samples shows the potential of this synthesis approach, it also opens the next challenge of growing single crystals with incorporated nominal *x* = 0.10 or higher concentration of Mg to perform a more in-debt study of the 9 T anomaly.

## Supporting Information Available

Rietveld refinement results, additional EDS data, low-temperature fits of the magnetic susceptibility, the EPR spectra of undoped and Mg-doped SrCu_2–*x*_Mg*_x_*(BO_3_)_2_ (with nominal *x* = 0.03, 0.05, and 0.10), schematics of a Cu^2+^ spin dimer lattice with two dimer-breaking impurity Mg^2+^ ions, and the calculated probability of doping dependence *P*_c_(x) for two Mg impurities located on two next-nearest dimers (PDF)
