# Supplementary material for: Higher-Magnesium-Doping Effects on the Singlet Ground State of the Shastry–Sutherland SrCu2(BO3)2
Source: Inorg Chem. 2024 Oct 16;63(43):20335–46. doi: 10.1021/acs.inorgchem.4c02398 (PMC11523215; doi:10.1021/acs.inorgchem.4c02398)
Supplement: Supplementary file 1 — ic4c02398_si_001.pdf [file ic4c02398_si_001.pdf]

Supplementary Information File

Higher-Magnesium-Doping Effects on the Singlet Ground State of the Shastry-Sutherland $SrCu_2(BO_3)_2$

Lia Šibav[1,2], Žiga Gosar[1,3], Tilen Knaflič[1,4], Zvonko Jagličić[5,6], Graham King[7], Hiroyuki Nojiri[8], Denis Arčon[1,3], and Mirela Dragomir[1,2*]

[1]Jožef Stefan Institute, Jamova cesta 39, 1000 Ljubljana, Slovenia
[2]Jožef Stefan International Postgraduate School, Jamova cesta 39, 1000 Ljubljana, Slovenia
[3]Faculty of Mathematics and Physics, University of Ljubljana, Jadranska ulica 19, 1000 Ljubljana, Slovenia
[4]Institute for the Protection of Cultural Heritage of Slovenia, Research Institute, Poljanska cesta 40, 1000 Ljubljana, Slovenia
[5]Institute of Mathematics, Physics and Mechanics, Jadranska ulica 19, 1000 Ljubljana, Slovenia
[6]Faculty of Civil and Geodetic Engineering, University of Ljubljana, Jamova cesta 2, 1000 Ljubljana, Slovenia
[7]Canadian Light Source, 44 Innovation Blvd, Saskatoon, SK S7N 2V3, Canada
[8] Institute for Materials Research, Tohoku University, Katahira 2-1-1, Sendai, 980-8577 Japan

*Corresponding author: mirela.dragomir@ijs.si

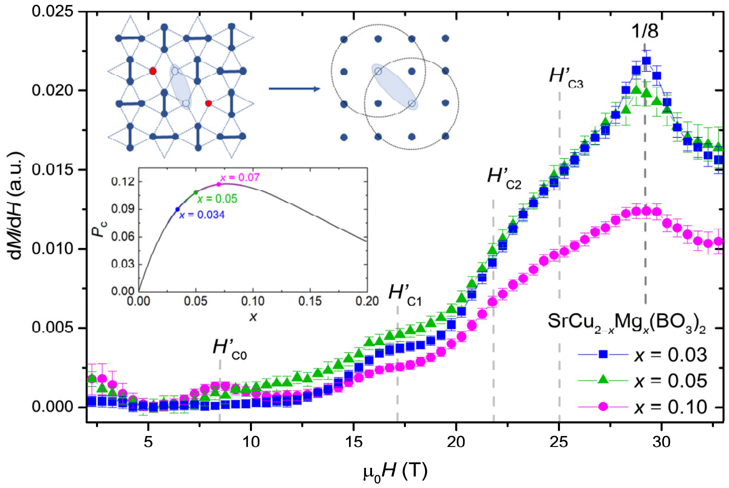

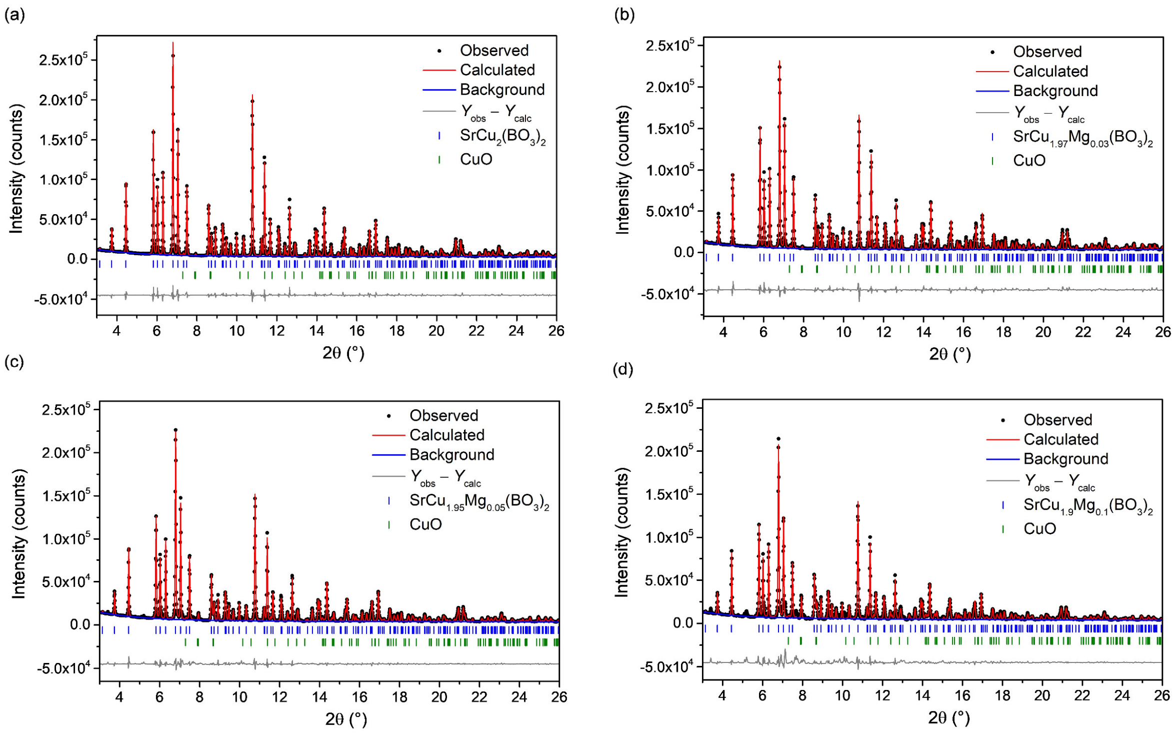

Figure S1. Profile fits of $SrCu_{2-x}Mg_x(BO_3)_2$ with x = 0 **(a)**, 0.03 **(b)**, 0.05 **(c)**, and 0.10 **(d)** using the $I\bar{4}2m$ space group. The synchrotron PXRD data (λ = 0.3502 Å) was collected at room-temperature.

Table S1. Structural parameters derived from the Rietveld refinements of $SrCu_{2-x}Mg_x(BO_3)_2$ with x = 0, 0.03, 0.05, and 0.10, at room temperature with $I\bar{4}2m$ space group. A systematic increase in the unit cell parameters a, b and c and the unit cell volume is observed with increasing Mg-doping concentration.

	$SrCu_2(BO_3)_2$ (x = 0)	$SrCu_{1.97}Mg_{0.03}(BO_3)_2$ (x = 0.03)	$SrCu_{1.95}Mg_{0.05}(BO_3)_2$ (x = 0.05)	$SrCu_{1.9}Mg_{0.1}(BO_3)_2$ (x = 0.1)
***a*, *b* (Å)**	8.9932(1)	8.9961(1)	8.9966(2)	8.9985(3)
***c* (Å)**	6.6509(1)	6.6552(1)	6.6566(2)	6.6584(3)
**V (Å^3)**	537.91(2)	538.60(2)	538.79(3)	539.15(5)
**R_{wp} (%)**<br>**R_{exp} (%)**	5.29<br>2.26	6.02<br>2.39	5.45<br>2.27	10.49<br>3.16
**$S = R_{wp}/R_{exp}$**	2.34	2.52	2.94	3.32
**Atom**	x/y/z<br>Occ.<br>$U_{11}/U_{22}/U_{33}$<br>$U_{12}/U_{13}/U_{23}$	x/y/z<br>Occ.<br>$U_{11}/U_{22}/U_{33}$<br>$U_{12}/U_{13}/U_{23}$	x/y/z<br>Occ.<br>$U_{11}/U_{22}/U_{33}$<br>$U_{12}/U_{13}/U_{23}$	x/y/z<br>Occ.<br>$U_{11}/U_{22}/U_{33}$<br>$U_{12}/U_{13}/U_{23}$
**Sr**	0/0.5/0<br>0.996(3)<br>0.0085(1)/0.0051(1)/0.0099(8)<br>0/0/0	0/0.5/0<br>1.015(3)<br>0.0120/0.0050/0.0105<br>0/0/0	0/0.5/0<br>1.017(5)<br>0.0086(14)/0.0099(15)/0.0115(10)<br>0/0/0	0/0.5/0<br>0.985(9)<br>0.0073(27)/0.0082(29)/0.0131(21)<br>0/0/0
**Cu**	0.1148(7)/0.1148(7)/0.2776(3)<br>1.00*<br>0.0043/0.0043/0.0278<br>0.0003/0.0027/0.0027	0.1149(9)/0.1149(9)/0.2799(2)<br>0.985*<br>0.0055/0.0055/0.0201<br>0.0050/0.0076/0.0076	0.1143(1)/0.1143(1)/0.2788(3)<br>0.975*<br>0.0023(6)/0.0023(6)/0.0315(16)<br>0.0006(12)/0.0014(16)/0.0014(16)	0.1140(2)/0.1140(2)/0.2798(6)<br>0.950*<br>0.0024/0.0024/0.0344<br>0.0008/0.0012/0.0012
**Mg**	-<br>-<br>-<br>-	0.1149(9)/0.1149(9)/0.2799(2)<br>0.015*<br>0.0055/0.0055/0.0201<br>0.0050/0.0076/0.0076	0.1143(1)/0.1143(1)/0.2788(3)<br>0.025*<br>0.0294/0.0294/0.0281<br>0.0050/0.0050/0.0050	0.1140(2)/0.1140(2)/0.2798(6)<br>0.050*<br>0.0050/0.0050/0.0072<br>0.0050/0.0050/0.0050*
**B**	0.2963(7)/0.2963(7)/0.2507(28)<br>1.012(15)<br>0.0080/0.0080/0.0120<br>0.0050/0.0050/0.0050	0.2944(8)/0.2944(8)/0.2329(27)<br>1.058(19)<br>0.0080/0.0080/0.0120<br>0.0050/0.0050/0.0050	0.2950(9)/0.2950(9)/0.254(4)<br>1.006(20)<br>0.0080/0.0080/0.0120<br>0.0050/0.0050/0.0050	0.2923(19)/0.2923(19)/0.257(7)<br>1.010(4)<br>0.0122/0.0122/0.0080<br>0.0106/0.0050/0.0050
**O1**	0.4019(4)/0.4019(4)/0.2126(11)<br>1.00*<br>0.0095/0.0095/0.0304<br>0.050/0.0050/0.0050	0.4010(5)/0.4010(5)/0.2079(13)<br>0.967(11)<br>0.0090/0.0090/0.0399<br>0.0021/0.0050/0.0050	0.4016(5)/0.4016(5)/0.2096(15)<br>1.015(14)<br>0.0136/0.0136/0.0192<br>0.0050/0.0039/0.0039	0.4015(11)/0.4015(11)/0.2086(26)<br>0.992(27)<br>0.0142/0.0142/0.0146<br>0.0120/0.0050/0.0050
**O2**	0.3274(3)/0.1440(3)/0.2523(13)<br>1.00*<br>0.0090/0.0080/0.0180<br>0.0010/0.0010/0.0020	0.3261(4)/0.1446(4)/0.2594(13)<br>1.072(7)<br>0.0121/0.0197/0.0097<br>0.0033/0.0050/0.0050	0.3263(4)/0.1446(4)/0.2515(18)<br>1.048(8)<br>0.0140/0.0143/0.0146<br>0.0032/0.0021/0.0050	0.3270(8)/0.1435(8)/0.256(3)<br>1.001(16)<br>0.0086/0.0050/0.0050<br>0.0010/0.0050/0.0017

* These parameters were fixed to the nominal values during the refinement.

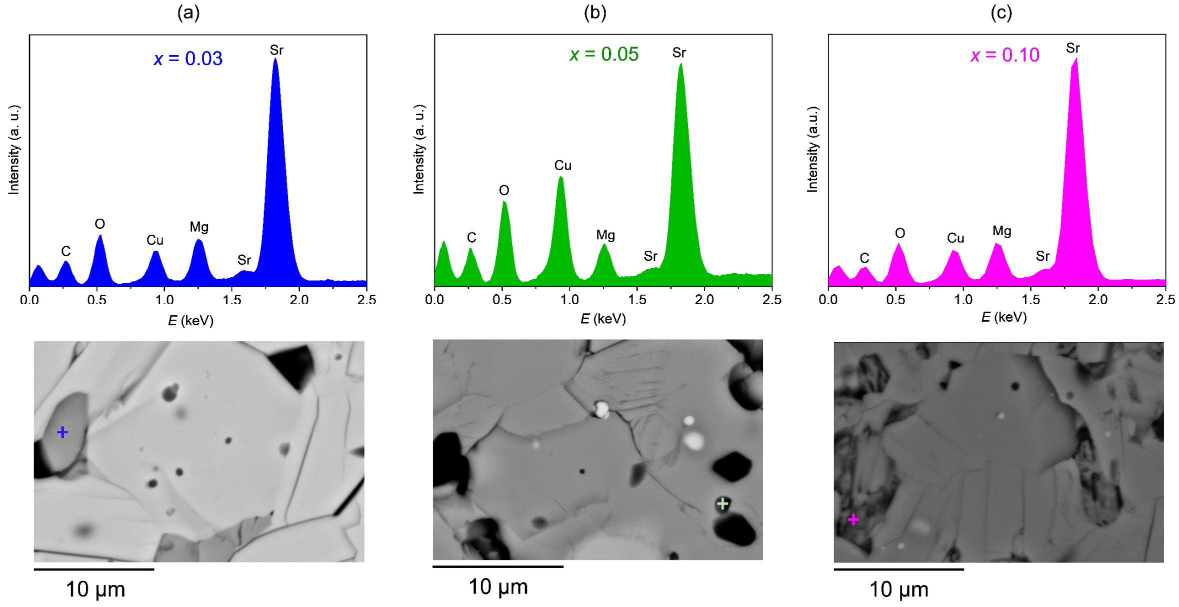

Figure S2. Normalised EDS point analysis spectra collected on the impurity sites for $SrCu_{2-x}Mg_x(BO_3)_2$, with $x = 0.03$, **(a)**, $x = 0.05$ **(b)** and $x = 0.10$ **(c)**. A higher concentration of Mg is suggested from the impurity sites compared to the doped $SrCu_2(BO_3)_2$ matrix. The corresponding SEM images can be seen below the EDS spectra.

Table S2. The complete results of magnetic susceptibility data fits in the temperature interval 2–6 K for different doping concentrations using the equation for total susceptibility at low temperatures (**Equation 5** in the main text). Due to a high correlation between the fitted parameters C' and θ', the fitting was performed using multiple fixed θ' values between 0 to −1, assuming antiferromagnetic interactions between the intrinsic impurities – dimer-free Cu^{2+} ions.

	C' (emu K/mol Cu)	χ_0 (emu/mol Cu)	θ′	Δ (K)	α (emu/mol Cu)
$SrCu_2(BO_3)_2$ $x = 0$	$1.72(2) \cdot 10^{-3}$	$2.37(6) \cdot 10^{-4}$	0	27.9(3)	$7.9(4) \cdot 10^{-2}$
	$2.07(1) \cdot 10^{-3}$	$1.72(5) \cdot 10^{-4}$	−0.25	27.2(2)	$7.2(2) \cdot 10^{-2}$
	$2.46(1) \cdot 10^{-3}$	$1.06(5) \cdot 10^{-4}$	−0.5	26.6(2)	$6.7(2) \cdot 10^{-2}$
	$2.88(2) \cdot 10^{-3}$	$4.0(5) \cdot 10^{-5}$	−0.75	26.1(1)	$6.3(1) \cdot 10^{-2}$
	$3.34(2) \cdot 10^{-3}$	$-2.7(5) \cdot 10^{-5}$	−1	25.6(2)	$5.9(1) \cdot 10^{-2}$
$SrCu_{1.97}Mg_{0.03}(BO_3)_2$ $x = 0.03$	$2.51(2) \cdot 10^{-3}$	$3.37(6) \cdot 10^{-4}$	0	24.1(2)	$4.2(1) \cdot 10^{-2}$
	$3.03(2) \cdot 10^{-3}$	$2.39(6) \cdot 10^{-4}$	−0.25	23.3(2)	$3.8(1) \cdot 10^{-2}$
	$3.61(2) \cdot 10^{-3}$	$1.40(6) \cdot 10^{-4}$	−0.5	22.6(2)	$3.55(9) \cdot 10^{-2}$
	$4.24(3) \cdot 10^{-3}$	$3.8(8) \cdot 10^{-5}$	−0.75	22.0(2)	$3.33(9) \cdot 10^{-2}$
	$4.92(4) \cdot 10^{-3}$	$-6(1) \cdot 10^{-5}$	−1	21.5(2)	$3.2(1) \cdot 10^{-2}$
$SrCu_{1.95}Mg_{0.05}(BO_3)_2$ $x = 0.05$	$3.69(2) \cdot 10^{-3}$	$4.95(9) \cdot 10^{-4}$	0	22.8(3)	$3.3(1) \cdot 10^{-2}$
	$4.48(2) \cdot 10^{-3}$	$3.41(7) \cdot 10^{-4}$	−0.25	21.5(2)	$2.81(8) \cdot 10^{-2}$
	$5.37(2) \cdot 10^{-3}$	$1.81(6) \cdot 10^{-4}$	−0.5	20.5(1)	$2.52(5) \cdot 10^{-2}$
	$6.35(3) \cdot 10^{-3}$	$1.7(8) \cdot 10^{-5}$	−0.75	19.6(2)	$2.32(5) \cdot 10^{-2}$
	$7.42(4) \cdot 10^{-3}$	$-1.5(1) \cdot 10^{-4}$	−1	18.9(2)	$2.18(4) \cdot 10^{-2}$
$SrCu_{1.9}Mg_{0.1}(BO_3)_2$ $x = 0.10$	$6.43(1) \cdot 10^{-3}$	$6.04(6) \cdot 10^{-4}$	0	21.0(2)	$2.20(6) \cdot 10^{-2}$
	$7.86(3) \cdot 10^{-3}$	$3.2(1) \cdot 10^{-4}$	−0.25	18.8(2)	$1.75(6) \cdot 10^{-2}$
	$9.48(6) \cdot 10^{-3}$	$1(2) \cdot 10^{-5}$	−0.5	17.1(3)	$1.51(7) \cdot 10^{-2}$
	$1.13(1) \cdot 10^{-2}$	$-3.1(3) \cdot 10^{-5}$	−0.75	15.9(4)	$1.38(6) \cdot 10^{-2}$
	$1.33(1) \cdot 10^{-2}$	$-6.5(5) \cdot 10^{-4}$	−1	14.9(4)	$1.31(6) \cdot 10^{-2}$

Table S3. The complete results of high-temperature magnetic susceptibility data fits in the interval 100–300 K for different doping concentrations using the Curie-Weiss law equation (**Equation 4** in the main text).

	C (emu K/mol Cu)	θ *(*K)	χ_0 (emu/mol Cu)
$SrCu_2(BO_3)_2$	0.553(4)	−136(1)	$-9.2(6) \cdot 10^{-5}$
$SrCu_{1.97}Mg_{0.03}(BO_3)_2$	0.517(2)	−133.3(7)	$-1.09(4) \cdot 10^{-4}$
$SrCu_{1.95}Mg_{0.05}(BO_3)_2$	0.480(2)	−129.9(6)	$-6.9(3) \cdot 10^{-5}$
$SrCu_{1.9}Mg_{0.1}(BO_3)_2$	0.379(2)	−110.1(9)	$2.1(4) \cdot 10^{-5}$

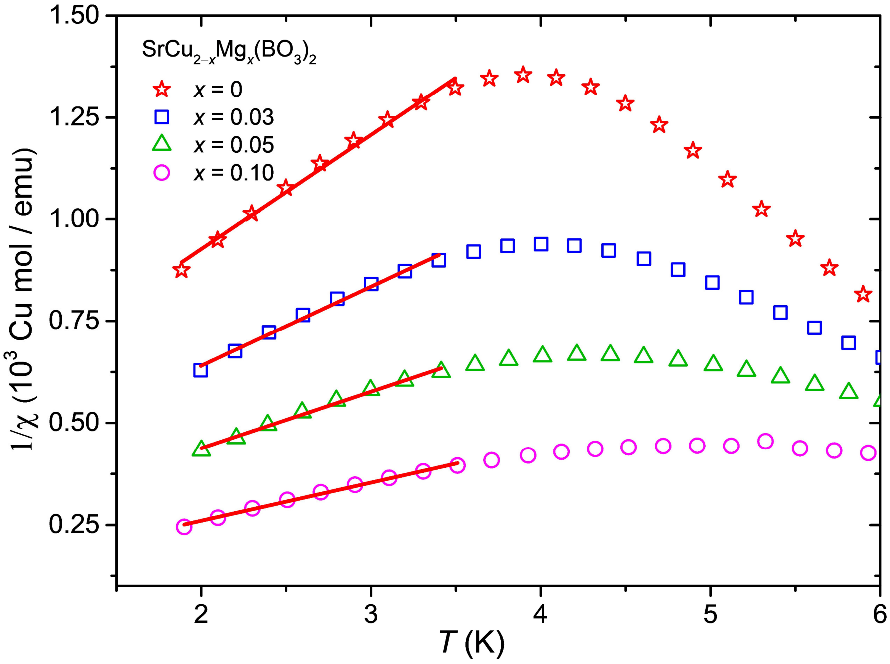

Figure S3. The low-temperature fits of the 1/χ data to **Equation 4** (main text) at T = 1.8–3.5 K for undoped and doped $SrCu_{2-x}Mg_x(BO_3)_2$ samples with x = 0, 0.03, 0.05, and 0.10. The concentration of Cu^{2+} S = 1/2 impurities was extracted from these fits for all doping concentrations.

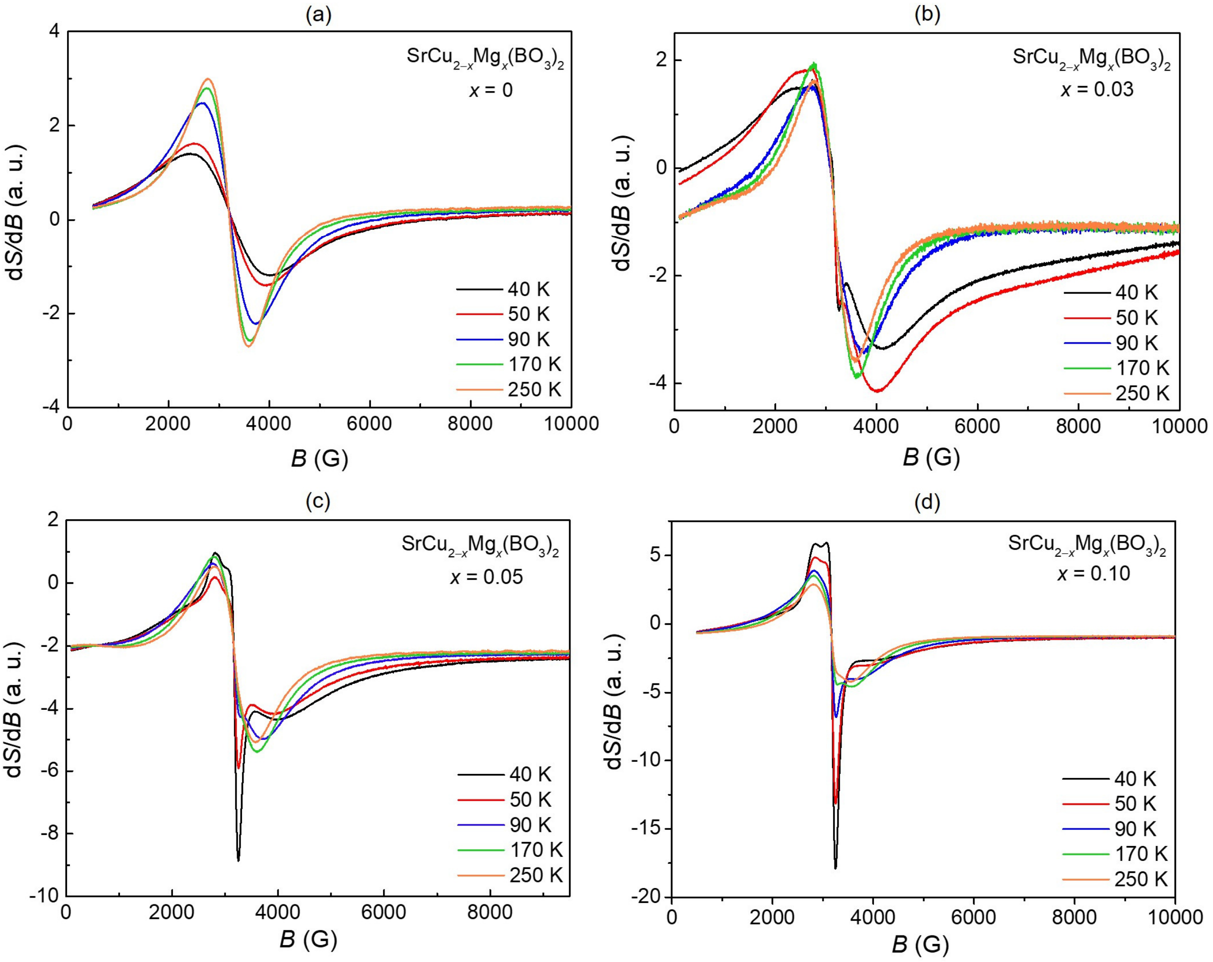

Figure S4. The EPR spectra of undoped $SrCu_2(BO_3)_2$ or $x = 0$ **(a)** and $SrCu_{2-x}Mg_x(BO_3)_2$ with $x = 0.03$ **(b)**, $x = 0.05$ **(c)**, and $x = 0.10$ **(d)** measured at temperatures of 40, 50, 90, 170, and 250 K, respectively, which show the development of low-temperature impurity peak that increases in intensity with increasing Mg-doping concentration.

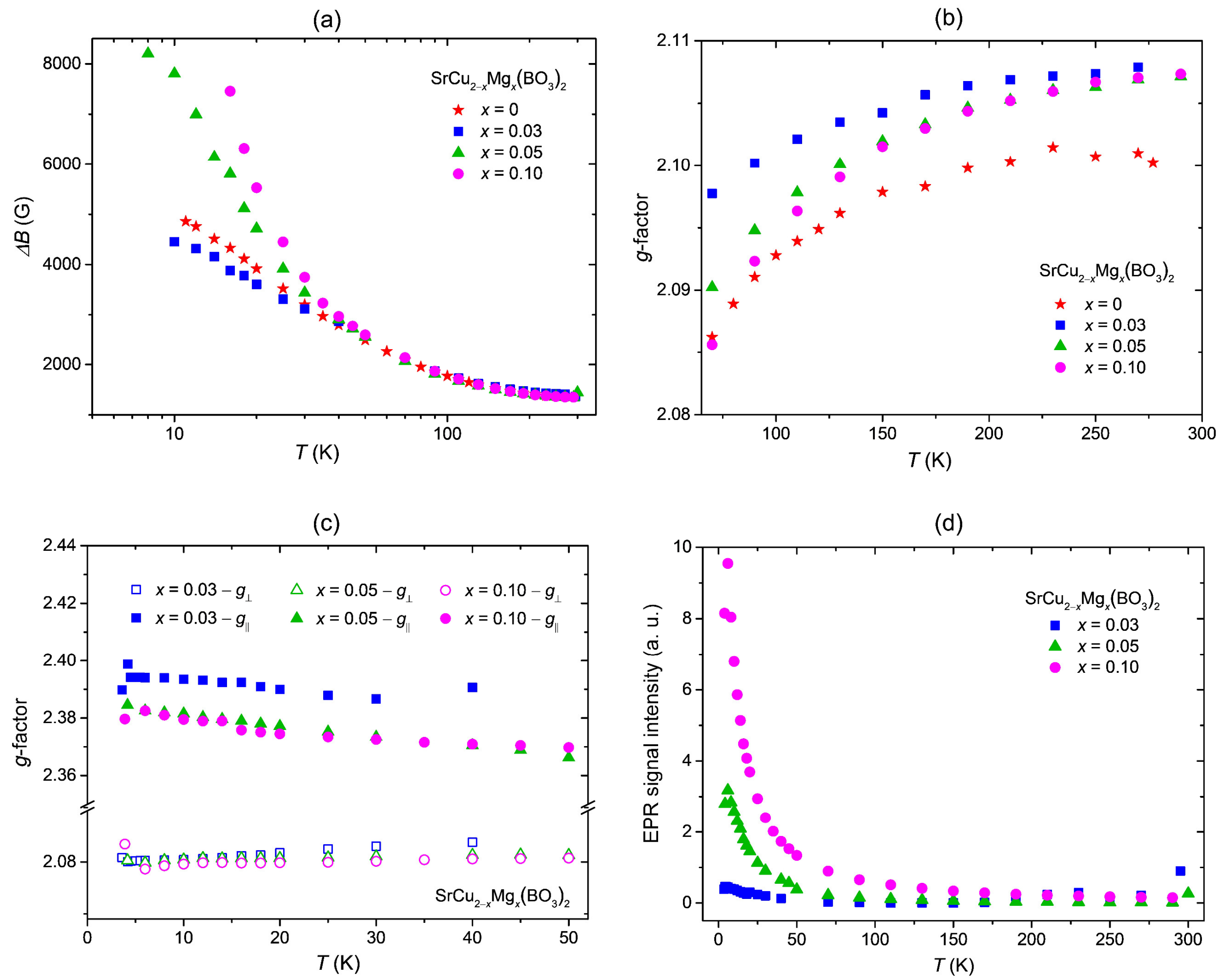

Figure S5. (a) The logarithmic temperature behaviour of the X-band EPR linewidth of the main dimer-lattice signal. **(b)** The temperature behaviour of the *g*-factor of the main dimer-lattice signal in $SrCu_{2-x}Mg_x(BO_3)_2$ with x = 0, 0.03, 0.05, and 0.10. **(c)** The temperature behaviour of the X-band EPR *g*-factors $g_{||}$ and $g_{\perp}$ as the two *g*- factor values along the *c* and *ab*-plane crystallographic directions for low-temperature "impurity" peaks for $SrCu_{2-x}Mg_x(BO_3)_2$ with x = 0.03, 0.05 and 0.10. **(d)** The temperature dependence of the X-band EPR signal intensity for low-temperature "impurity" peaks for x = 0.03, 0.05 and 0.10 samples.

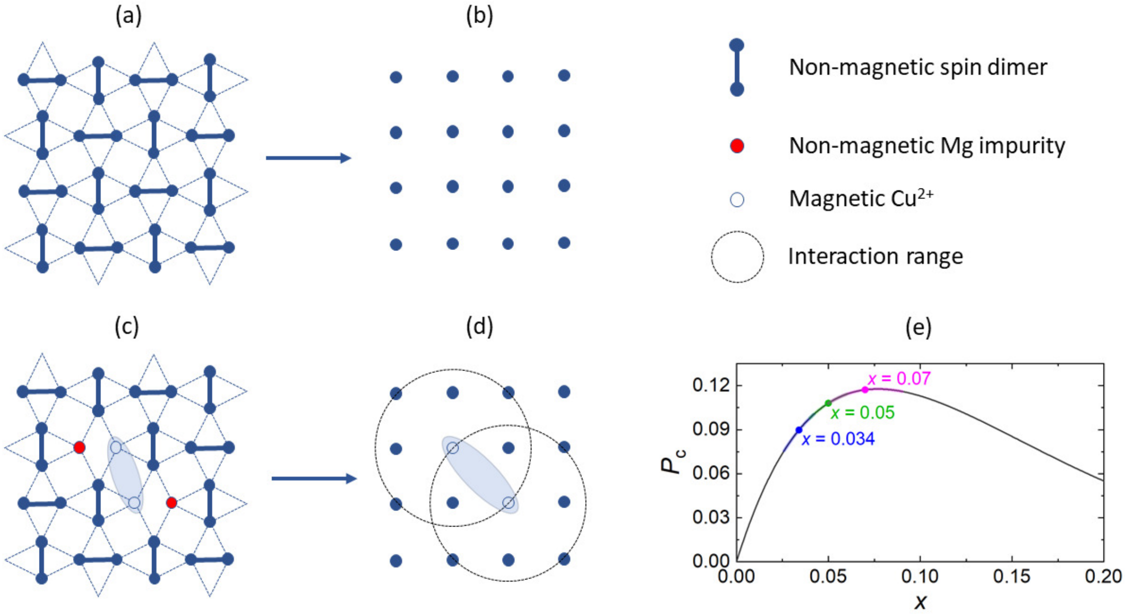

Figure S6. (a) Schematic image of Cu^{2+} spin dimer lattice, shown as two solid blue circles joined by a solid blue line. **(b)** Square lattice of Cu^{2+} spin dimers, where every lattice site marks the position of a centre of a dimer. Each dimer is represented by a solid blue circle. **(c)** Schematic image of a Cu^{2+} spin dimer lattice with two dimer-breaking impurity Mg^{2+} ions (solid red circles). Open blue circles show liberated magnetic Cu^{2+} spins. The shaded ellipse shows coupling of the two next-nearest-neighbouring magnetic Cu^{2+} spins. **(d)** Transformation of the spin dimer lattice with two impurities to the square lattice, similar as in (b). Solid blue circles show non-magnetic spin dimers and open blue circles magnetic Cu^{2+}. The shaded ellipse shows the case of coupling of two next-nearest-neighbouring Cu^{2+} and the dashed circles mark the interaction range for this case. **(e)** Calculated doping dependence of probability $P_c(x)$ for the formation of two next-nearest-neighbouring liberated magnetic Cu^{2+} spins as shown in (d). Blue, green and magenta points on the curve show the probability values for the experimentally determined doping x = 0.034, 0.05 and 0.07. Respective colour-shaded parts of the curve show the range of the uncertainty in x.

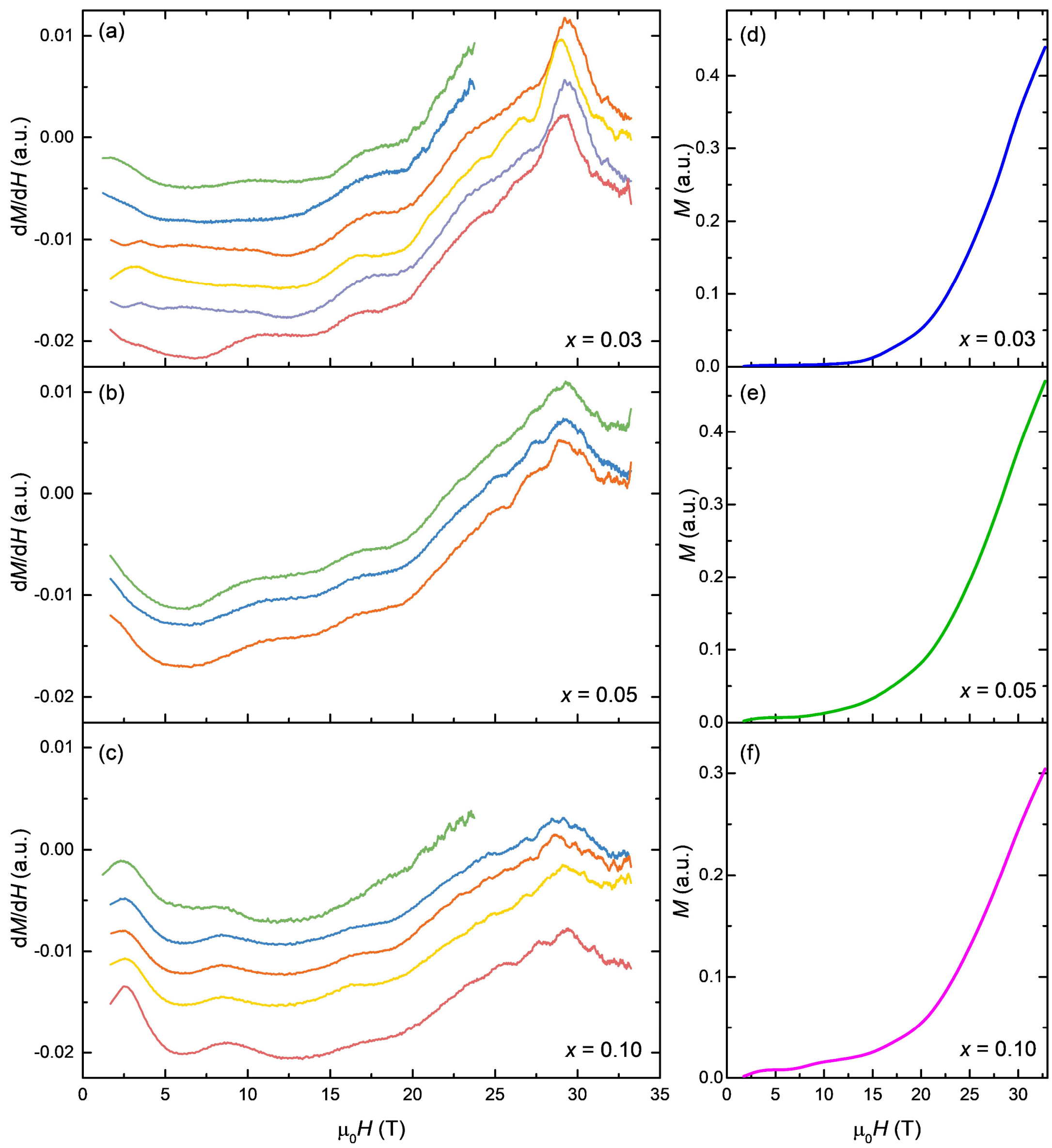

Figure S7. High magnetic field measurements performed in a pulsed magnetic field on $SrCu_{2-x}Mg_x(BO_3)_2$ powders. On the left the derivative d*M*/d*H* ($\mu_0 H$) is shown for *x* = 0.03 (**a**), 0.05 (**b**), and 0.10 (**c**). The derivative was numerically calculated from the time series of the raw magnetisation data and the magnetic field. A Savitzky-Golay filter with window length of 21 and a polynomial order of 1 (linear approximation) was applied for smoothing. The plots were vertically offset for clarity. The data for the lowest and highest 5% of magnetic fields reached during the pulse were omitted because the magnetic field changes slowly at these fields, resulting in higher errors. On the right side, the corresponding averaged magnetisation curves are shown for *x* = 0.03 (**d**), 0.05 (**e**), and 0.10 (**f**).
